# Quantitative Proteomic Analysis of BHK-21 Cells Infected with Foot-and-Mouth Disease Virus Serotype Asia 1

**DOI:** 10.1371/journal.pone.0132384

**Published:** 2015-07-10

**Authors:** Hui-Chen Guo, Ye Jin, Shi-Chong Han, Shi-Qi Sun, Yan-Quan Wei, Xian-Ji Liu, Xia Feng, Ding Xiang Liu, Xiang-Tao Liu

**Affiliations:** 1 State Key Laboratory of Veterinary Etiological Biology and OIE/National Foot and Mouth Disease Reference Laboratory, Lanzhou Veterinary Research Institute, Chinese Academy of Agricultural Sciences, Lanzhou, Gansu, China; 2 School of Biological Sciences, Nanyang Technological University, Singapore; 3 Guangzhou Fitgene Biotechnology Co. Ltd. Guangzhou International Business Incubator A110, Guangzhou, Guangdong, China; University of Hong Kong, HONG KONG

## Abstract

Stable isotope labeling with amino acids in cell culture (SILAC) was used to quantitatively study the host cell gene expression profile, in order to achieve an unbiased overview of the protein expression changes in BHK-21 cells infected with FMDV serotype Asia 1. The SILAC-based approach identified overall 2,141 proteins, 153 of which showed significant alteration in the expression level 6 h post FMDV infection (57 up-regulated and 96 down-regulated). Among these proteins, six cellular proteins, including three down-regulated (VPS28, PKR, EVI5) and three up-regulated (LYPLA1, SEC62 and DARs), were selected according to the significance of the changes and/or the relationship with PKR. The expression level and pattern of the selected proteins were validated by immunoblotting and confocal microscopy. Furthermore, the functions of these cellular proteins were assessed by small interfering RNA-mediated depletion, and their functional importance in the replication of FMDV was demonstrated by western blot, reverse transcript PCR (RT-PCR) and 50% Tissue Culture Infective Dose (TCID50). The results suggest that FMDV infection may have effects on the expression of specific cellular proteins to create more favorable conditions for FMDV infection. This study provides novel data that can be utilized to understand the interactions between FMDV and the host cell.

## Introduction

Foot-and-mouth disease (FMD) is one of the most economically important diseases of cloven-hoofed animals because it severely compromises livestock production, resulting in high economic losses and international restrictions on the export of animals and animal products [1].The causative agent is the foot-and-mouth disease virus (FMDV) belonging to genus *Aphthovirus* of the family *Picornaviridae*, which is a non-enveloped virus possessing positive polarity and a single-stranded RNA genome around 8kb in length. The viral RNA is translated into one large polyprotein that is post-translationally cleaved by viral proteinase into individual structural and nonstructural proteins. Four structural proteins, VP1, VP2, VP3, and VP4, form the natural empty capsid with 60 copies of each protein. Nonstructural proteins, including L^pro^, 2A, 2B, 2C, 3A, 3B, 3C^pro^ and 3D^pol^, regulate viral replication, protein processing, and protein modification in the host [1, 2]. During infection, the virus affects the host cell in multiple ways through viral proteins. Accordingly, viral proteins can be grouped into different functional categories. P1 protein, especially VP1, has a role in virus attachment. L^pro^, a papain-like proteinase, mediates autocatalytic cleavage from a polyprotein and is involved in modulating host response to infection by inhibiting the transcription of various immunological factors to antagonize the antiviral state of the host [3, 4]. 3C^pro^ can inhibit host cell transcription by cleavage of host proteins[5]. 2A is an autoproteinase involved in the cleavage of virus polyprotein[6, 7]. 2B, 2C and 3A proteins co-localize with the intracellular membrane system and may be related to viral RNA replication. 3B (VPg) can trigger virus genome replication by binding to the 5′ terminus of the genome [7]. 3Dpol interacts with 3A to form the viral replication complex and fulfill the function of a replicase[8].

FMDV infection results in multiple effects on the host cell, such as signaling pathways and morphology. During its whole life cycle, FMDV can interact with the host cell in several ways, ranging from activation of the innate immunity to establish an antiviral state in the cell, to utilizing cellular processes for efficient replication of FMDV[9–12]. Nevertheless, the pathogenesis of FMDV is not well understood at present. Novel insight into host-virus interactions gained from quantitative proteomic studies of the infected cells would drastically enhance our understanding of the FMDV infection processes, and may reveal novel cellular targets for the design of more effective antivirals and the development of new vaccine strategies. To this end, a global approach to investigate changes in the host cell proteome in response to FMDV infection, namely, stable isotope labeling with amino acids in cell culture (SILAC) was used in conjunction with LC–MS/MS in this study. The results showed that the abundance and expression patterns of 153 proteins were significantly changed upon FMDV infection. Among them, six proteins were selected and validated by using western blot, laser confocal microscopy and other functional assays. These studies confirmed there were significant alterations of cellular protein expression profile in FMDV-infected cells compared with mock-infected cells, and verified that variations in the expression of specific proteins would render differential effects on FMDV replication at different steps of the replication cycle, such as RNA replication, protein translation and virus titer.

## Materials and Methods

### Cells and virus

Baby hamster kidney 21 (BHK-21) from China Center for Type Culture Collection (CCTCC, Wuhan, China) were grown in Dulbecco’s modified Eagle’s medium (DMEM) (Invitrogen) supplemented with 10% fetal bovine serum (FBS; HyClone), 100 U/mL penicillin and 100 mg/mL streptomycin, at 37°C with 5% CO_2_. FMDV strain Asia1/Jiangsu/China/2005 (GenBank Accession No. EF149009) was preserved at OIE/National Foot-and-Mouth Disease Reference Laboratory (Lanzhou, Gansu, P.R China) and propagated for 10 passages in BHK-21 cells.

### Isotopic labeling in cell culture

DMEM was supplemented with 10% dialyzed fetal calf serum and all of the lacking amino acids (Invitrogen), except for L-lysine. The medium was then divided and supplemented with L-Lysine-2HCl or [U-^13^C_6_]-L-Lysine (Invitrogen) to produce light or heavy SILAC medium, respectively. BHK-21 cells were grown in parallel in both media, and passage was routinely performed every 2 to 3 days (as appropriate) at 80% to 90% confluence. After a minimum of seven population doublings, the cells were analyzed by mass spectrometry (MS) and achieved virtually complete incorporation of heavy L-lysine (S1 and S2 Figs).

### Infection

Heavy-labeled cells were infected with FMDV, and BHK-21 cells cultured in medium with conventional amino acid source were used as mock-infected controls. Cells passaged in the two media were seeded in 75 cm^2^ cell culture flasks until they reached 75% confluence. The heavy-labeled cells were then infected with FMDV at a multiplicity of infection (MOI) of 1 and the light-labeled cells were mock-inoculated. Meanwhile, the dialyzed serum in the SILAC media was decreased to 2%. Cells were harvested at 6 hours post-infection (h.p.i.) using cell lysis buffer.

### Gel electrophoresis and in-gel digestion

The total protein concentrations in the cell lysates were quantified and equivalent amounts of cell proteins from two independent biological replicates were diluted in SDS-PAGE loading buffer containing 5% β-mercaptoethanol prior to boiling, separated using one-dimensional SDS-PAGE (4% to 12% Novex Bis-Tris mini gel, Invitrogen), and then visualized by Coomassie blue stain (Pierce). The entire protein gel lane was excised in parallel and cut into 48 gel slices each. The gel slices were subsequently subjected to in-gel digestion with trypsin (20 ng/μL) at 37°C overnight, as described previously [13, 14]. The resulting tryptic peptides were extracted by 2.5% formic acid and 90% acetonitrile, lyophilized in a SpeedVac (Helena Biosciences), and resuspended in 0.1% formic acid and 2% acetonitrile before LC–MS/MS analysis.

### LC–MS/MS

LC–MS/MS analysis was performed by Guangzhou Fitgene Biotechnology Co., Ltd., as described previously [14, 15]. In brief, the gel was cut into 48 slices from which proteins were digested and consequent peptides were extracted and then lyophilized before further analysis. The powder of peptides was resuspended in solvent A (2% acetonitrile, 0.1% formic acid in water) and loaded onto the C18 reverse phase column (100 μm in diameter, 15 cm long, 3 μm resin from Michrom Bioresources, Auburn, CA). Each mixture of peptide was separated with a linear gradient of solvent B from 5% to 15% within 15 minutes followed by a gradient from 15% to 35% over 85 minutes, end and sustain at 90% for the following 20 minutes. Eluted peptides were injected directly to LTQ-Obitrap XL (Thermo Fisher Scientific, Inc.) through a nanoelectrospray ion source (ProxeonBiosystems) with a voltage of 1.85kV and a temperature of transfer capillary of 200°C. Data-dependent mode was employed to acquire data using Xcalibur software (Thermo Electron). An accumulation of 10^6^ ions was required to trigger a MS full scan, with maximal accumulation time of 500 ms and resolution of 60000 (m/z 400), ranging from 400–2000 Dalton. Six most intensive ions per MS scan were selected and fragmented by CID in LTQ to perform the MSMS scan with an accumulation of at least 5000 ions and the maximal accumulation time of 100 ms. The normalized collision energy was 35%, activation Q was 0.25, activation time was 30 ms, dynamic exclusion was enable with a maximum retention period of 90 s and a relative mass window of 10 ppm. The lock mass (PCM, MW445.12) was introduced to improve mass accuracy in survey scan.

### Quantification, bioinformatics and protein analysis

Quantification was carried out using MaxQuant version [1.0.13.13], based on two-dimensional centroid of the isotope clusters within each SILAC pair, as previously described [14, 15]. Protein ratios were calculated as the median of all SILAC pair ratios belonging to peptides in the protein. The derived peak list was searched using the Mascot search engine (version 2.1.04; Matrix Science, London, UK) against the NCBI Cricetidae database under taxonomy ID 337677 (Date:2012-01-09,Total number of sequence:64093) and the decoy database (reverse database). The normalized H/L ratios, significance, and variability (%) were automatically produced by the MaxQuant 1.0.13.13 program. The resulting proteingroups.txt output file contained the peptide identification was imported into Microsoft Excel. The threshold of acceptance was set at false discovery rate (FDR) 1% at both peptide and protein level. Both unique and razor peptides included in the protein group were used for quantification with minimal ratio count of 1 (the ratio of protein group was calculated once a spectra with quantitative ratio was available). Ingenuity Pathways Analysis (Ingenuity Systems, www.ingenuity.com) was used to establish probable interaction networks of altered proteins, based on published reports and protein interaction databases. Data sets containing gene identifiers and corresponding expression values were used to generate the networks. Each gene in Ingenuity Pathways Knowledge Base was matched to the corresponding gene identifier.

### Western blot analysis

Total protein concentrations in cell lysates prepared from FMDV-infected and uninfected cells harvested at 6 h p.i. were measured, and equivalent amounts of cell lysates from two independent biological replicates were denatured by heating at 100°C for 10 min in 5×sample loading buffer and separated using 10% SDS-PAGE. Proteins were electrotransferred to 0.45 μm nitrocellulose membranes (Bio-Rad), blocked with 5% nonfat milk in TBS containing 0.05% Tween-20 (TBST) overnight at 4°C. The membranes were then incubated for 1 h at room temperature with specific antibodies against DARS (Santa Cruz Biotechnology, Inc., USA), LYPLA1 (Abcam, UK), SEC62 (Sigma-Aldrich, USA), EIF2AK2 (Abcam, UK), EVI5 (Sigma-Aldrich, USA),VPS28 (Sigma-Aldrich, USA), Tublin (Santa Cruz Biotechnology, Inc., USA)or β-actin (Santa Cruz Biotechnology, Inc., USA). Incubation with horseradish peroxidase (HRP)-conjugated secondary antibodies (Sigma) was performed at room temperature for 1 h, followed by washing with TBST three times. The protein bands were visualized using Amersham ECL Plus Western Blot Detection Reagents (GE Healthcare).

### Immunofluorescence assay

Coverslip-adhered BHK-21 cell monolayers were infected with FMDV at an MOI of 1. The plates were inoculated at 37°C over 1 h followed by replacement with fresh growth media, and were incubated at 37°C for another 5 h. The cells were fixed with 4% PFA and treated with 0.01% Triton X-100, with the following antibodies applied: DARS (Santa Cruz Biotechnology, Inc., USA), LYPLA1 (Abcam, UK), SEC62 (Sigma-Aldrich, USA), EIF2AK2 (Abcam, UK), EVI5 (Sigma-Aldrich, USA),VPS28 (Sigma-Aldrich, USA), or β-actin (Santa Cruz Biotechnology, Inc., USA). FMDV proteins were detected by pig anti-FMDV Asia 1 VLPs serum (primary antibody, 1:100) recognized by a TRITC-conjugated secondary antibody (Sigma). DAPI was used as counterstain to allow visualization of cell nuclei. Immunofluorescence confocal microscopy images were captured on an LSM510 META microscope (Carl Zeiss, Ltd.).

### siRNA experiment

Three sets of siRNA per gene targeting three different sites within the coding region were purchased from Santa Cruz Biotechnology, Inc., USA. BHK-21 cells were seeded into six-well plates and transfected with Oligofectamine (Invitrogen) in a final concentration of 30 nM siRNA. In each experiment, siRNA universal negative controls (Sigma) were used as controls. The cells were then inoculated with FMDV at an MOI of 1 at 30 h after siRNA transfection. The cells were then washed and fresh culture medium was added 1 h after virus inoculation. Afterward, cells were obtained to assess the presence of infectious FMDV particles using Western blot, RT–PCR (reverse transcription–PCR), and 50% Tissue Culture Infective Dose (TCID_50_) at 6 h p.i. Each siRNA experiment was performed in triplicate.

### TCID50 detection of FMDV after RNA interference

The TCID_50_ in the collected supernatants was determined by virus titration assay, as described previously [16]. Briefly, a BHK-21 cell suspension in DMEM with 5% FBS at a concentration of 1.5×10^6^ cells/mL was dispensed at 50 μL per well into 96-well flat-bottomed tissue culture plates. The plates were rocked to achieve uniform suspension thickness and incubated at 37°C for 24 h to 36 h under 5% CO_2_ tension to attain 90% confluence. Serial 10-fold dilutions of virus stock prepared in FBS-less DMEM were added in 50 μL volumes to all wells. Plates were incubated at 37°C, 5% CO_2_ for 72 h, and the presence or absence of cytopathic effect (CPE) was then monitored. TCID_50_ was calculated by Reed–Muench method.

### RT–PCR analyses of FMDV gene

Reverse transcription PCR analyses of FMDV gene post-RNA interference were performed to quantify FMDV RNA in BHK-21 cells. Total RNA was extracted using Trizol reagent (Ambion) according to the manufacturer’s protocol. Reverse primers for FMDV 3D or β-actin were used to synthesize cDNA fragments by using reverse transcriptase M-MLV (TaKaRa). Subsequently, PCR was performed using *rTaq* polymerase (TaKaRa) and specific primers for either FMDV 3D or β-actin (FMDV 3D primers, forward: 5-TTCGGCCTTTGATGCTAACCACTG-3, reverse: 5-GCATCCCGCCCTCAACAACAAT-3; β-actin primers, forward: 5-CGGCATCCACGAAACTAC-3, reverse: 5-ATCTTCATCGTGCTGGGCG-3). For replication of FMDV 3D gene or β-actin gene, the amplification program was set at 94°C for 25 s, 56°C for 25 s, 72°C for 20 sec for 20 cycles. The sizes and uniqueness of PCR products were verified by agarose gel electrophoresis.

### Statistical analysis

The data of relative quantity in RT-PCR, western blot and TCID_50_ are presented as mean ± SD after analysis by Image J software. The statistical analysis of variance between groups was performed by SPSS Statistics 19.0 software. One-way ANOVA Comparison between groups using Least Significance Difference (LSD) was applied. Significant difference of all statistical tests was set at 0.05 (p < 0.05).

## Results

### Optimal time-point for collection of BHK-21 cells infected with FMDV serotype Asia 1

**Fig 1:**
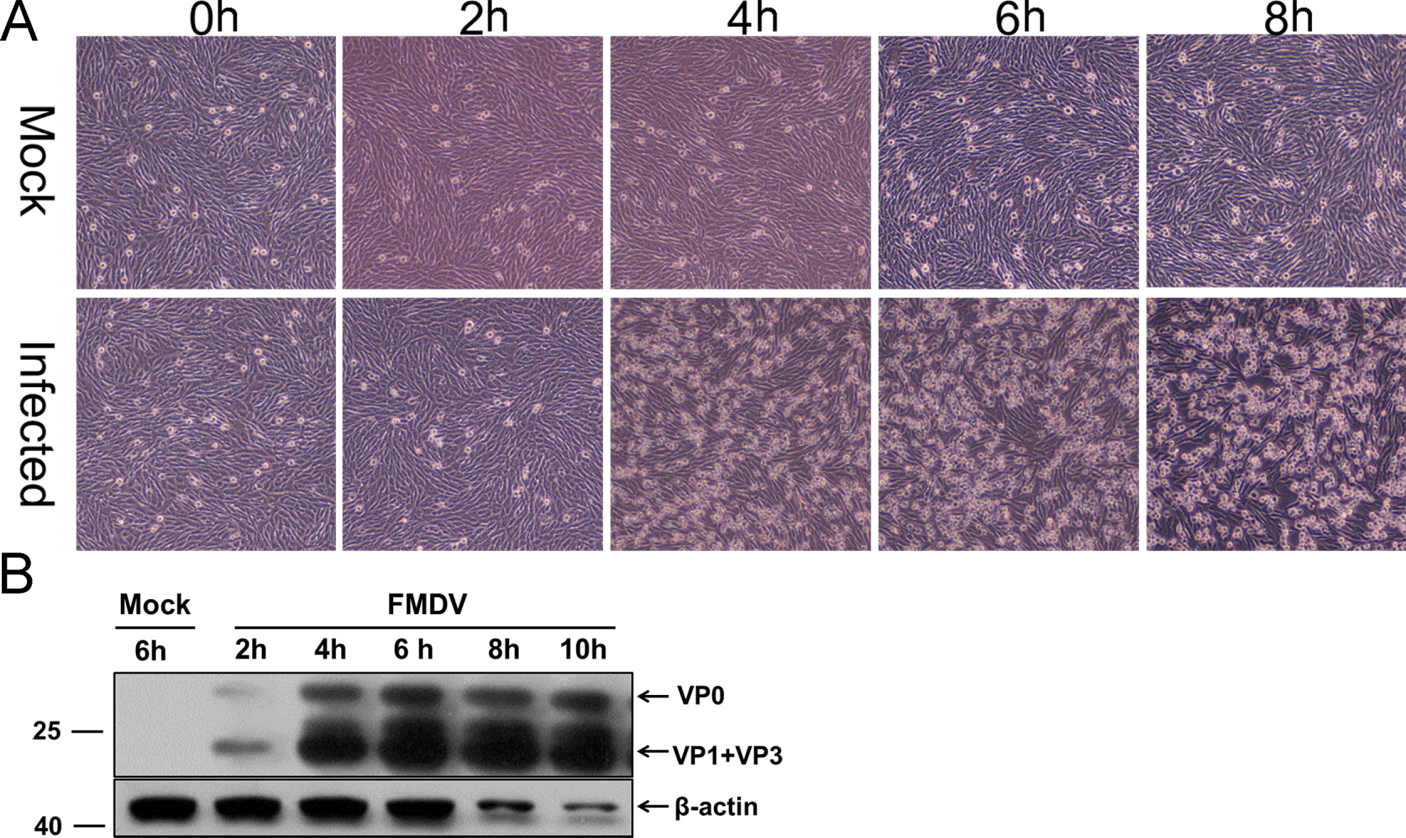
FMDV infection in BHK-21 cells. (A) Photomicrographs of BHK-21 cells infected with FMDV serotype Asia1 at MOI = 1 PFU/cell or mock-infected for the indicated hours post infection (h.p.i.). Images were taken at an original magnification of 100×. (B) Confirmation of FMDV capsid proteins in the cell lysate at the indicated h.p.i. by western blot using the pig anti-FMDV Asia 1 serum as primary antibody. Actin was used as loading control.

A distinctive feature in FMDV-infected BHK-21 cells is the formation of CPE, which denotes a virus-induced alteration of cells including general stress responses, especially cell death. Dead lytic cells plus FMDV-induced suppression of host cell protein synthesis cause a drastic decrease in the quantity of many cellular proteins, at times reaching undetectable. Thus, to ascertain a time-point for maximal effect with minimal negative effect of CPE after infection of FMDV, BHK-21 cells were infected with FMDV serotype Asia 1 at an MOI of 1 and microscopically monitored for CPE. Afterward, the FMDV protein was detected by Western blot against pig anti-FMDV Asia 1 serum over time. As shown in Fig 1A, CPE appeared at approximately 4 h p.i. and was readily observed at later time points. Consistently, the capsid protein of FMDV increased over time (Fig 1B). Although the FMDV protein expression reached the peak at 8h p.i., the expression of β-actin as a control was markedly decreased at this time point, suggesting virus-induced suppression of host cell protein synthesis and lysis or death of a majority of the cells at 8h p.i. Therefore, combining the results of CPE and the expression of FMDV and cellular proteins, we chose to examine the composition of cells at 6 h p.i. in the subsequent studies, at this time point the expression of virus structural proteins was just initialized and cellular proteins were kept stable.

### SILAC coupled with LC–MS/MS and bioinformatics analyses of FMDV-infected BHK-21 cells

**Fig 2:**
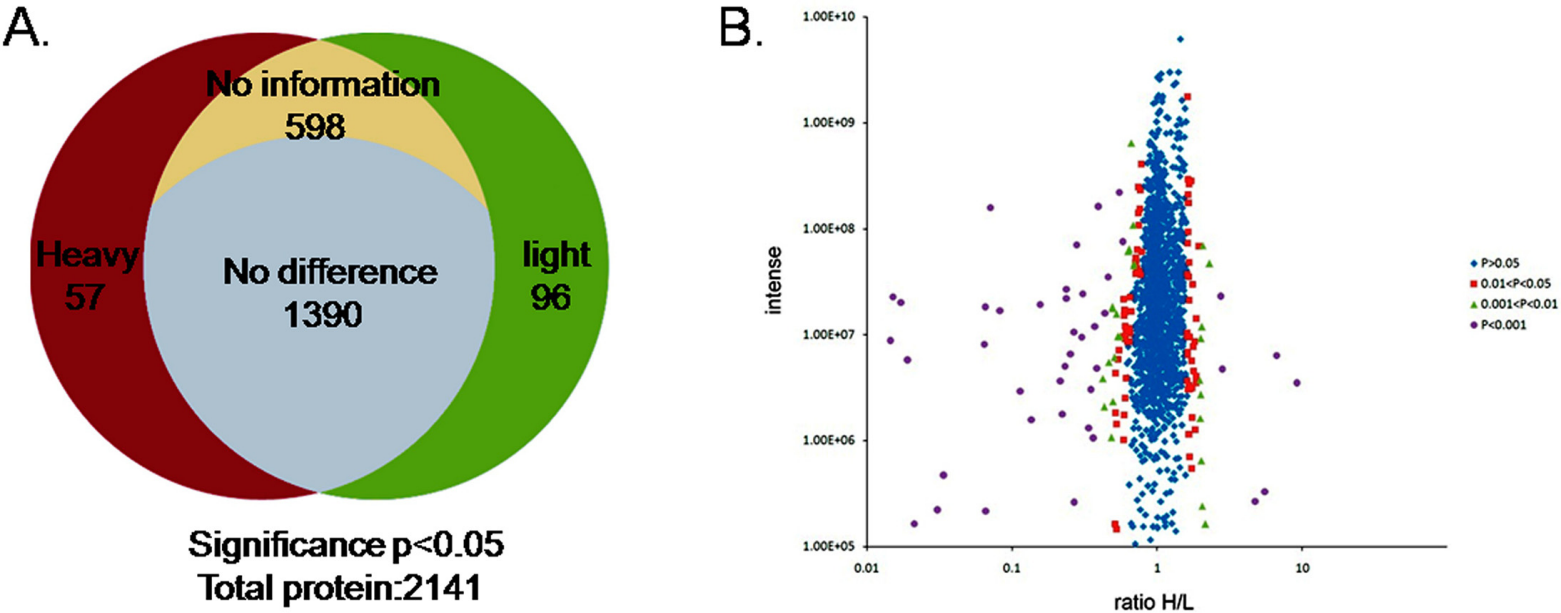
Distributions of proteins identified in BHK-21 cells infected with FMDV and proteome-wide accurate quantitation and significance. (A) The left (black red color) represents up-regulated host proteins in virus-infected cells. The right (green color) represents down-regulated host proteins based on significance B ≤ 0.05. The other parts represent unchanged proteins in FMDV-infected cells. (B) Signal intensities (log10) of all quantified proteins in the FMDV-infected cells are shown as functions of their fold change (log2). The spread of the cloud is lower at high abundance, indicating that quantification is more precise. The criterion of being identified as a significantly regulated protein can be evaluated by the significance B level indicated in blue, red, purple, and green.

Although a previous paper used SILAC coupled with LC–MS/MS to identify and quantify proteome changes in IB-RS-2 cells infected with serotype O FMDV was published [15], no research related to quantitative proteomics of BHK-21 cells infected with FMDV serotype Asia 1 was available before this study. In this study, we obtained cellular proteomes from BHK-21 cells, known as the universal cell line for FMDV proliferation and production of FMD vaccine, and compared the differences in the levels of the isolated proteins by quantitative proteomics for the first time. Mock-infected cells were grown in media labeled with lightlysine medium, and cells infected with FMDV serotype Asia 1 at an MOI of 1 were grown in media containing heavy 13C6 HCl-Lys. Cells were collected at 6 h.p.i. Equal amounts of proteins were subsequently mixed, identified and quantified. For quantification, by setting the threshold at 0.05 in significance B, which was calculated from log protein ratios as a measurement of outlier significance score, proteins with values less than 0.05 were defined as significantly varied members and added to the candidate pool for investigating potential proteome changes between data sets through IPA (S1 Table). LC–MS/MS analysis identified 2,141 proteins in whole cellular fractions, which were identified by two or more peptides. Among these, 57 and 96 proteins were identified and quantified to be up-regulated and down-regulated at a significance B value≤0.05, as calculated by MaxQuant, respectively (Tables 1 and 2). Meanwhile, no significant change in abundance was observed for 1,390 proteins (64.92%), and 598 proteins (27.93%) could not be quantified (Fig 2).

**Table 1:** Proteins present in increased abundance in FMDV-infected cells.

Accession number	Protein description	Protein name	Ratio (infection /control)	Peptides	Functions
gi|354485463	membrane-associated progesterone receptor component 2-like	PGRMC2	9.20	2	Receptor for steroids
gi|344254339	Aspartyl-tRNA synthetase	DARS	6.70	1	aminoacyl-tRNA ligase activity
gi|344249005	Translocation protein SEC62	SEC62	5.52	2	transporter activity
gi|354488911	acyl-protein thioesterase 1-like	LYPLA1	4.75	3	translation elongation factor activity
gi|354475980	DNA ligase 1-like	LIG1	2.82	1	ATP binding/DNA binding
gi|354494845	lamina-associated polypeptide 2	TMPO	2.75	17	DNA binding
gi|344257723	Serine/arginine repetitive matrix protein 2	SRRM2	2.35	1	RNA splicing
gi|354481274	dihydrolipoyllysine-residue succinyltransferase component of 2-oxoglutarate dehydrogenase comple	DLST	2.30	2	transferase activity, transferring acyl groups
gi|344244946	Lipase maturation factor 2	LMF2	2.16	4	endoplasmic reticulum membrane
gi|344246379	Cancer-associated gene 1 protein-like	SSR1	2.06	4	Endoplasmic reticulum
gi|354481751	glypican-4	GPC4	2.04	1	glycoprotein binding
gi|344253619	Legumain	LGMN	2.04	4	endopeptidase activity
gi|354465940	disks large homolog 1 isoform 1	DLG1	2.02	2	iron ion binding
gi|125204	cAMP-dependent protein kinase catalytic subunit alpha	PRKACA	2.00	1	protein kinase activity
gi|344245500	Muscle-related coiled-coil protein	MURC	2.00	1	Activator/Developmental protein
gi|354475251	neutral cholesterol ester hydrolase 1-like	NCEH1	1.99	1	carboxylesterase activity
gi|344251417	Putative deoxyribose-phosphate aldolase	DERA	1.97	5	Catalyzes a reversible aldol reaction between acetaldehyde and D-glyceraldehyde 3-phosphate to generate 2-deoxy-D-ribose 5-phosphate
gi|344258664	Metalloproteinase inhibitor 1	TIMP1	1.93	5	enzyme inhibitor activity
gi|354485259	synaptobrevin homolog YKT6-like	YKT6	1.86	2	endoplasmic reticulum membrane
gi|354488481	dnaJ homolog subfamily A member 3	DNAJA3	1.86	2	purine nucleotide binding
gi|354485509	mitochondrial import receptor subunit TOM70-like	TIMM70A	1.86	1	receptor activity
gi|354489198	eukaryotic initiation factor 4A-III-like	EIF4A3	1.83	3	translation initiation factor activity
gi|354467882	ATP synthase mitochondrial F1 complex assembly factor 2-like	ATPAF2	1.82	15	May play a role in the assembly of the F1 component of the mitochondrial ATP synthase
gi|354478986	metaxin-1-like	MTX1	1.80	3	Involved in transport of proteins into the mitochondrion
gi|4126964	peroxisomal membrane anchor protein	PEX14	1.76	1	protein N-terminus binding
gi|153581939	glycerolphosphate dehydrogenase	GPD2	1.75	12	glyceraldehyde-3-phosphate dehydrogenase (NAD+) (phosphorylating) activity
gi|354506683	sorting and assembly machinery component 50 homolog	SAMM50	1.75	1	May be required for the assembly pathway of mitochondrial outer membrane proteins
gi|354482314	5'-AMP-activated protein kinase catalytic subunit alpha-1-like	PRKAA1	1.74	6	protein kinase activity
gi|344255846	BAG family molecular chaperone regulator 3	BAG3	1.73	1	chaperone binding
gi|354497226	integrin alpha-5	ITGA5	1.73	1	receptor activity
gi|1730177	Glucose-6-phosphate isomerase	GPI	1.72	5	glucose-6-phosphate isomerase activity
gi|354498914	delta-1-pyrroline-5-carboxylate dehydrogenase	ALDH4A1	1.68	1	1-pyrroline-5-carboxylate dehydrogenase activity
gi|344251256	Annexin A11	ANXA11	1.67	1	calcium ion binding
gi|344249997	Mitochondrial import inner membrane translocase subunit Tim17-B	TIMM17B	1.67	2	transporter activity
gi|2499845	CD44 antigen	CD44	1.67	1	Receptor
gi|344237837	Mitochondrial 2-oxoglutarate/malate carrier protein	SLC25A11	1.67	1	signal transducer activity
gi|354485616	hydroxymethylglutaryl-CoA lyase	HMGCL	1.67	7	lyase activity
gi|354474977	SRA stem-loop-interacting RNA-binding protein	SLRIP	1.66	8	RNA binding
gi|124426	Inosine-5'-monophosphate dehydrogenase 2	IMPDH2	1.66	1	DNA binding
gi|354488213	citrate synthase, mitochondrial-like	CS	1.66	1	transferase activity, transferring acyl groups
gi|354494828	protein NipSnap homolog 2-like	GBAS	1.65	7	mitochondrion/Inferred from sequence or structural similarity
gi|344245669	Prohibitin	PHB	1.65	16	sequence-specific DNA binding RNA polymerase II transcription factor activity
gi|354498001	endoplasmic reticulum metallopeptidase 1-like	ERMP1	1.65	9	peptidase activity
gi|327248646	ATP synthase alpha subunit 1	ATP5A1	1.64	4	receptor binding
gi|344248533	Electron transfer flavoprotein subunit beta	ETFB	1.64	2	electron carrier activity
gi|344259127	GPI transamidase component PIG-S	PIGS	1.63	1	Component of the GPI transamidase complex
gi|354469583	complement component 1 Q subcomponent-binding protein	C1QBP	1.63	4	Binds to the globular "heads" of C1Q thus inhibiting C1 activation
gi|344249404	Prostacyclin synthase	PTGIS	1.63	2	monooxygenase activity
gi|344257987	Isocitrate dehydrogenase	IDH2	1.62	1	isocitrate dehydrogenase activity
gi|344245224	Annexin A5	ANXA5	1.62	2	calcium ion binding
gi|344243386	ATP synthase subunit delta, mitochondrial	ATP2D	1.62	2	transporter activity
gi|344236740	Succinyl-CoA:3-ketoacid-coenzyme A transferase 1	OXCT1	1.62	3	3-oxoacid CoA-transferase activity
gi|354483129	ras-related protein Rab-12-like	RAB12	1.61	5	GTP binding
gi|344243359	Basigin	BSG	1.61	2	mannose binding
gi|15721885	calnexin	CANX	1.61	1	calcium ion binding
gi|344245614	Cytochrome b-c1 complex subunit 2	UQCRC2	1.60	9	metalloendopeptidase activity

**Table 2:** Proteins present in decreased abundance in FMDV-infected cells.

Accession number	Protein description	Protein name	Ratio (infection /control)	Peptides	Functions
gi|334303194	eukaryotic translation initiation factor 2 alpha kinase 2 variant 2kinase 2	EIF2AK2	0.01	4	translation factor activity, nucleic acid binding
gi|354488450	hypothetical protein LOC100769587	hypothetical protein	0.01	7	No report
gi|354488340	peroxisomal carnitine O-octanoyltransferase isoform 2	CROT	0.02	2	transferase activity, transferring acyl groups
gi|344240087	Protein KRI1-like	KRI1	0.02	15	No report
gi|344251862	EVI5-like protein	EVI5	0.02	6	small GTPase regulator activity
gi|354474640	MAP7 domain-containing protein 2	MAP7D2	0.02	1	No report
gi|344236597	Vacuolar protein sorting-associated protein 28-like	VPS28	0.03	3	Component of the ESCRT-I complex, a regulator of vesicular trafficking process
gi|354500438	potassium voltage-gated channel subfamily D member 3-like	KCND3	0.03	1	transporter activity
gi|344242595	Dolichyl-diphosphooligosaccharide—protein glycosyltransferase subunit DAD1	DAD1	0.06	7	dolichyl-diphosphooligosaccharide-protein glycotransferase activity
gi|344237874	Anionic trypsin-2	PRSS2	0.07	12	peptidase activity
gi|354486039	DDB1- and CUL4-associated factor 4-like	DCAF4L1	0.07	6	No report
gi|354493412	cullin-5-like isoform 1	CUL5	0.07	2	enzyme binding
gi|354478266	NEDD8 ultimate buster 1 isoform 2	NUB1	0.08	1	Specific down-regulator of the NEDD8 conjugation system
gi|354475105	KN motif and ankyrin repeat domain-containing protein 2	KANK2	0.11	1	guanyl-nucleotide exchange factor activity
gi|354466146	plastin-1	PLS1	0.14	10	No report
gi|344254912	Cleavage stimulation factor 50 kDa subunit	CSTF1	0.16	5	One of the multiple factors required for polyadenylation and 3'-end cleavage of mammalian pre-mRNAs
gi|17376715	Golgi apparatus protein 1	GLG1	0.22	20	receptor binding
gi|354473428	immunoglobulin-like and fibronectin type III domain-containing protein 1-like	lrfn1l	0.22	1	May be involved in the regulation of excitatory synapses
gi|344236075	Integrin alpha-1	ITGA1	0.23	3	receptor activity
gi|344237623	Scaffold attachment factor B1	SAFB	0.24	1	small molecule binding
gi|354477755	ectopic P granules protein 5 homolog	EPG5	0.24	1	Involved in autophagy. May play a role in the degradation step of the autophagy pathway
gi|354473176	inverted formin-2-like	INF2	0.25	6	cytoskeletal protein binding
gi|344243254	U4/U6.U5 tri-snRNP-associated protein 1	SART1	0.27	1	May play a role in mRNA splicing. May also bind to DNA.
gi|344249797	Phosphatidylinositol-binding clathrin assembly protein	PICALM	0.27	2	phospholipid binding
gi|354485371	small acidic protein-like	-	0.28	42	No report
gi|344238413	Phosphoacetylglucosamine mutase	PGM3	0.30	24	magnesium ion binding
gi|344251937	Signal transducer and activator of transcription 3	STAT3	0.31	3	signal transducer activity
gi|354507834	calcium-binding and coiled-coil domain-containing protein 1-like	CALCOCO1	0.34	9	chromatin binding
gi|154243355	stearoyl-CoA desaturase 1	SCD1	0.35	8	stearoyl-CoA 9-desaturase activity
gi|344257067	Coiled-coil domain-containing protein 6	CUL3	0.36	7	Core component of multiple cullin-RING-based BCR (BTB-CUL3-RBX1) E3 ubiquitin-protein ligase complexes which mediate the ubiquitination and subsequent proteasomal degradation of target proteins
gi|354472580	sarcoplasmic/endoplasmic reticulum calcium ATPase 2-like	ATP2A2	0.37	1	transporter activity
gi|28194647	cathepsin L	CTSL1	0.38	23	peptidase activity
gi|354470621	myosin-3-like	MYH3	0.39	3	cytoskeletal protein binding
gi|354468695	calcium/calmodulin-dependent protein kinase type II subunit gamma-like	CAMK2G	0.42	1	protein kinase activity
gi|354504107	endothelial differentiation-related factor 1-like	EDF1	0.43	2	sequence-specific DNA binding
gi|354507394	myosin regulatory light chain 2	MYH2	0.44	1	calcium ion binding
gi|344238999	Isopentenyl-diphosphate Delta-isomerase 1	IDI1	0.46	2	hydrolase activity
gi|344247197	putative E3 ubiquitin-protein ligase TRIP12	TRIP12	0.47	5	ligase activity
gi|354468967	regulatory-associated protein of mTOR	PRTOR	0.48	8	Involved in the control of the mammalian target of rapamycin complex 1 (mTORC1) activity which regulates cell growth and survival
gi|354496956	TAR DNA-binding protein 43-like isoform 2	TARDBP	0.49	7	small molecule binding
gi|354465282	E3 ubiquitin-protein ligase NEDD4-like	NEDD4	0.49	1	ligase activity
gi|354482078	exocyst complex component 4	EXOC4	0.50	7	Component of the exocyst complex involved in the docking of exocytic vesicles with fusion sites on the plasma membrane
gi|344237164	RNA-binding protein 7	RBM7	0.51	1	small molecule binding
gi|344240655	Integrin beta-3	ITGB3	0.51	5	receptor activity
gi|344242040	Uncharacterized protein C1orf77-like	C11orf58	0.51	8	No report
gi|344258772	40S ribosomal protein S6	Rps6	0.52	1	structural molecule activity
gi|344249426	GTPase HRas	HRAS	0.52	2	GTP binding
gi|354471711	microtubule-associated protein 1A-like	MAP1A	0.52	2	Structural protein involved in the filamentous cross-bridging between microtubules and other skeletal elements.
gi|354481682	C-type mannose receptor 2	MRC2	0.53	3	receptor activity
gi|354478904	ubiquitin-associated protein 2 isoform 1	UBAP2L	0.54	2	binding of sperm to zona pellucida
gi|344250619	Protein FAM49A	FAM49A	0.54	1	Inferred from electronic annotation
gi|344239224	Vigilin	HDLBP	0.55	1	RNA binding
gi|344257977	putative ATP-dependent RNA helicase DDX17	DDX17	0.55	1	helicase activity
gi|354494465	developmentally-regulated GTP-binding protein 1	DRG1	0.56	6	GTP binding
gi|354498518	suppressor of G2 allele of SKP1 homolog	SUGT1	0.58	6	No report
gi|354483672	spectrin beta chain	SPTB	0.58	4	cytoskeletal protein binding
gi|354476738	lysosomal protective protein isoform 1	CTSA	0.59	3	carboxypeptidase activity
gi|344256627	DNA-directed RNA polymerase II subunit RPB3	POLR2C	0.59	2	DNA-directed RNA polymerase activity
gi|354495341	stathmin-like	STMN1	0.59	23	Involved in the regulation of the microtubule (MT) filament system by destabilizing microtubules
gi|354502497	cleavage and polyadenylation specificity factor subunit 7 isoform 1	CPSF7	0.59	1	small molecule binding
gi|344254882	Maspardin	SPG21	0.60	4	May play a role as a negative regulatory factor in CD4-dependent T-cell activation
gi|354479041	cell division cycle 5-related protein-like	CDC5L	0.60	1	DNA binding
gi|344243055	putative ATP-dependent RNA helicase DDX5	DDX5	0.60	8	helicase activity
gi|354488191	SAP domain-containing ribonucleoprotein-like	Syncrip	0.60	16	No report
gi|354470481	caprin-1-like	CAPRIN1	0.60	4	RNA binding
gi|354504568	DNA-directed RNA polymerase II subunit RPB7-like	RPB7	0.61	3	DNA-directed RNA polymerase activity
gi|354473311	40S ribosomal protein S3-like	RPS3	0.61	1	structural molecule activity
gi|354498985	EF-hand domain-containing protein D2-like	EFHD2	0.61	1	calcium ion binding
gi|354499471	spectrin alpha chain, brain-like isoform 2	SPTAN1	0.63	7	Actin capping
gi|344237085	U4/U6.U5 tri-snRNP-associated protein 2	USP39	0.63	4	ubiquitin thiolesterase activity
gi|354468104	protein transport protein Sec23B isoform 1	SEC23B	0.64	1	DNA-directed RNA polymerase activity
gi|344247943	40S ribosomal protein S3a	RPS3	0.64	1	structural molecule activity
gi|344254132	Dynamin-like 120 kDa protein	OPA1	0.65	10	GTPase activity
gi|344250281	Claudin-3	CLDN3	0.65	1	structural molecule activity
gi|344236900	Pyridoxal-dependent decarboxylase domain-containing protein 1	PDXDC1	0.65	5	lyase activity
gi|354483820	guanine nucleotide-binding protein subunit beta-2-like 1-like	GNB2L1	0.66	1	ion channel inhibitor activity
gi|354492355	eukaryotic translation initiation factor 4 gamma 2-like	EIF4G2	0.66	2	translation initiation factor activity
gi|344255980	Cullin-3	Cul3	0.69	12	enzyme binding
gi|354473860	regulator of nonsense transcripts 1-like	GL50803_13452	0.69	2	ATP binding
gi|344253625	Actin-related protein 2/3 complex subunit 2	ARPC2	0.69	2	as actin-binding component of the Arp2/3 complex which is involved in regulation of actin polymerization and together with an activating nucleation-promoting factor (NPF) mediates the formation of branched actin networks.
gi|354486221	long-chain-fatty-acid—CoA ligase 4-like	ACSL4	0.71	5	ligase activity
gi|354506341	protein transport protein Sec31A	SEC31A	0.71	2	protein transport
gi|344239124	Protein transport protein Sec23A	Sec23A	0.71	1	zinc ion binding
gi|344251913	Eukaryotic translation initiation factor 1	EIF1	0.72	1	translation initiation factor activity
gi|20373098	RNA helicase	RNA-H	0.72	3	sequence-specific DNA binding transcription factor activity
gi|354493469	transcription factor BTF3-like isoform 2	BTF3	0.73	9	transcription coactivator activity
gi|49652	40S ribosomal protein S24	RPS24	0.74	1	structural constituent of ribosome
gi|354476231	coatomer subunit alpha isoform 2	COPA	0.74	3	structural molecule activity
gi|344255737	40S ribosomal protein S26	RPS26	0.75	23	structural constituent of ribosome
gi|344248597	Programmed cell death 6-interacting protein	PDCD61P	0.75	6	microtubule organizing center
gi|354497705	40S ribosomal protein S12-like	RPS12	0.76	3	structural constituent of ribosome
gi|344240144	Eukaryotic translation initiation factor 2 subunit 3	EIF2	0.77	4	translation initiation factor activity
gi|354488129	nascent polypeptide-associated complex subunit alpha-like	Naca	0.77	2	DNA binding
gi|1083173	carbohydrate-binding protein CBP30	LGALS3	0.78	6	sugar binding
gi|344258446	60S ribosomal protein L23a	RPL23A	0.78	1	Structural constituent of ribosome

**Fig 3:**
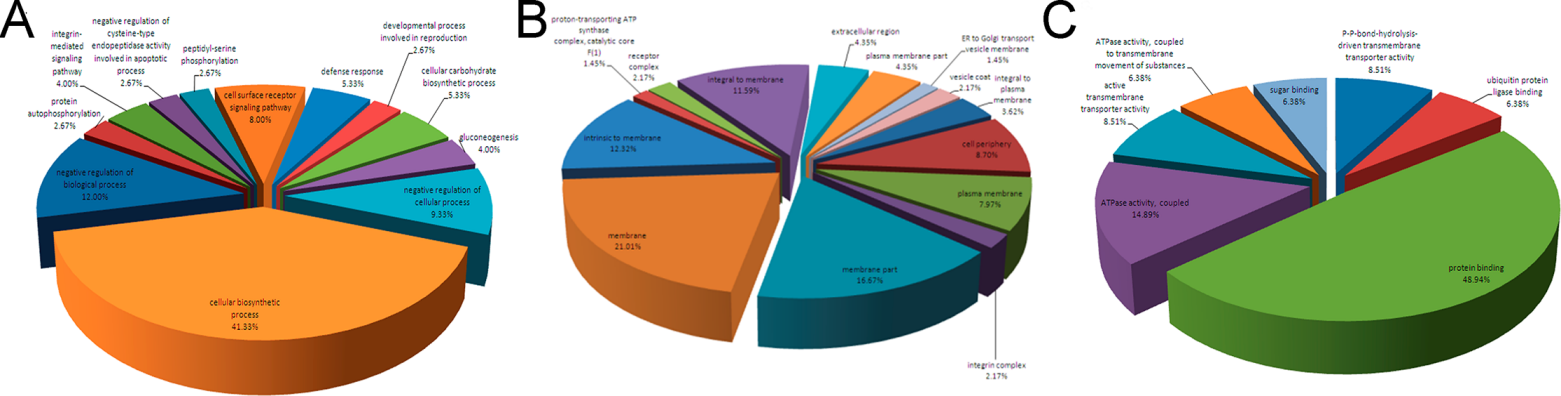
Classification of proteins in BHK-21 cells infected with FMDV according to their assigned fraction and biological function. (A) Biological process; (B) Cellular component; (C) Molecular functions. More information is available in Supplemental material (S1 Table and S3 Fig).

Based on the underlying biology evidence from the UniProtKB/Swiss-Prot, TrEMBL protein databases and the Gene Ontology database, 163 identified proteins were assigned to different molecular functional classes and subcellular annotations, in order to gain functional insights into the cellular proteome. The hamster genome database had poor annotation compared with the mouse genome, so the number of identified proteins was limited. To avoid missing information, gene identifications of proteins were converted to mouse protein GI numbers. Based on their underlying biological evidence from the literature database, the interacting pathways were constructed by importing the protein GI numbers and levels of regulation into the Ingenuity Pathways Analysis (IPA) tool. The underlying biological implication of the varied proteins was deciphered by enriching the annotations of Gene Ontology (GO) possessed by these proteins. The enrichment was conducted with GO miner in three main aspects comprising Biological Process (BP), Cellular Component (CC), and Molecular Function (MF) (Fig 3). In FMDV-infected cells, the up-regulated proteins are found to be mainly associated with cellular responses to stimuli, protein binding, localization and transport; whereas the down-regulated proteins are mainly related to the nucleotide and nucleoside activities, catabolic and hydrolase functions, alternative splicing, and cell adhesion. Fig 3 shows the relative proportion of proteins in each separate functional class (also see S3 Fig).

### Network pathway analysis

**Fig 4:**
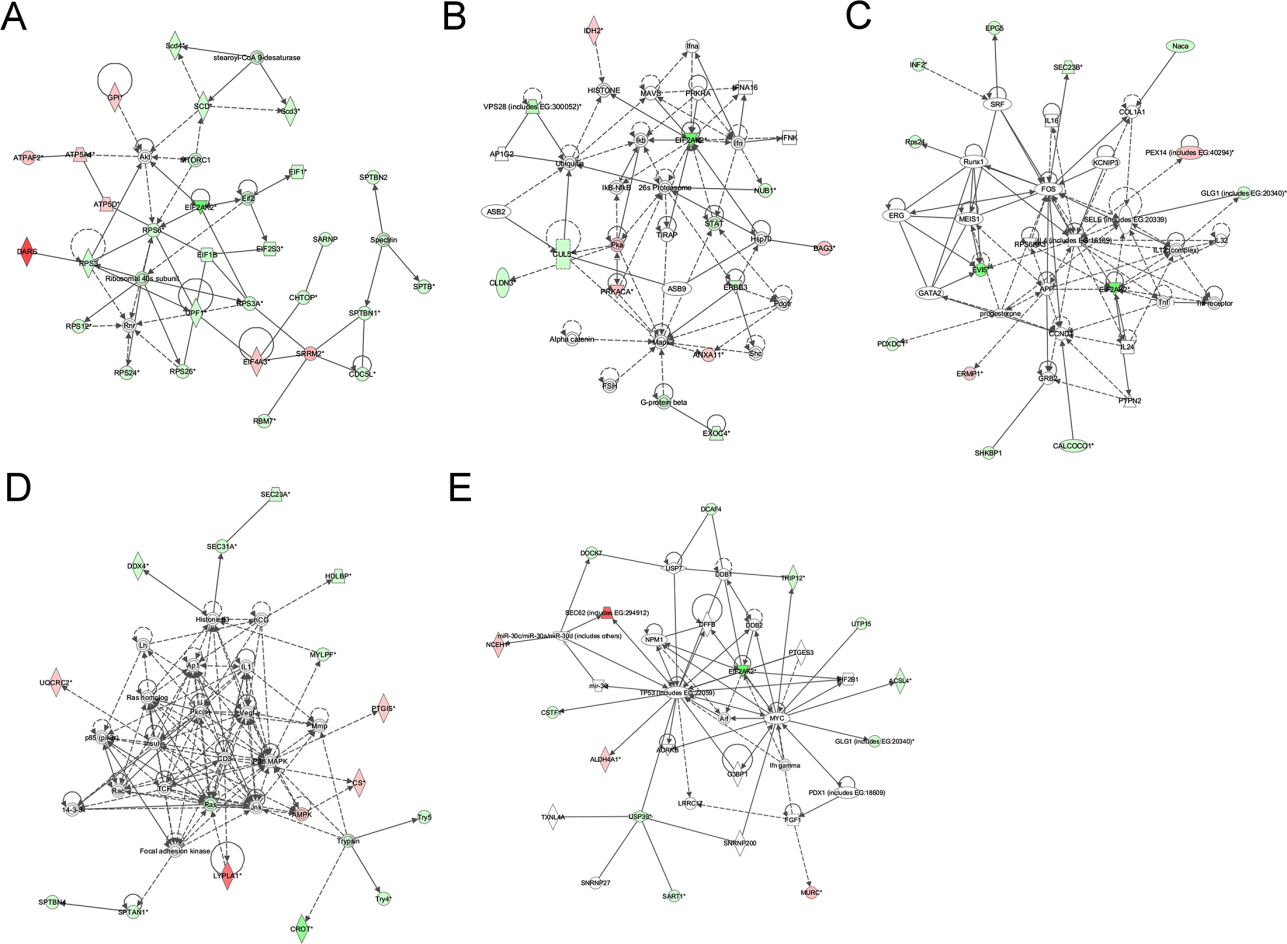
Ingenuity pathway analysis of proteins that were significantly altered in BHK-21 cells infected with FMDV Asia 1. The proteins indentified were involved in Protein Synthesis, Gene Expression, Nucleic Acid Metabolism (A); Cell Death, Inflammatory Response, Infectious Disease (B); Hematological System Development and Function, Hematopoiesis, Cellular Development (C); Gene Expression, Cell Death, Cell Morphology (D); DNA Replication, Recombination and Repair, Cancer, Skeletal and Muscular Disorders (E). Proteins shaded in green indicate decrease in abundance and proteins shaded in red indicate increase in abundance in the FMDV-infected cells compared with mock-infected cells. The color intensity corresponds to the degree of abundance. Proteins in white are those known to be in the network but were not identified in our study through the Ingenuity Pathways Knowledge Base. The shapes denote the molecular class of the protein. The solid lines indicate the promotional interactions while the dashed lines indicate the suppressive interaction.

**Fig 5:**
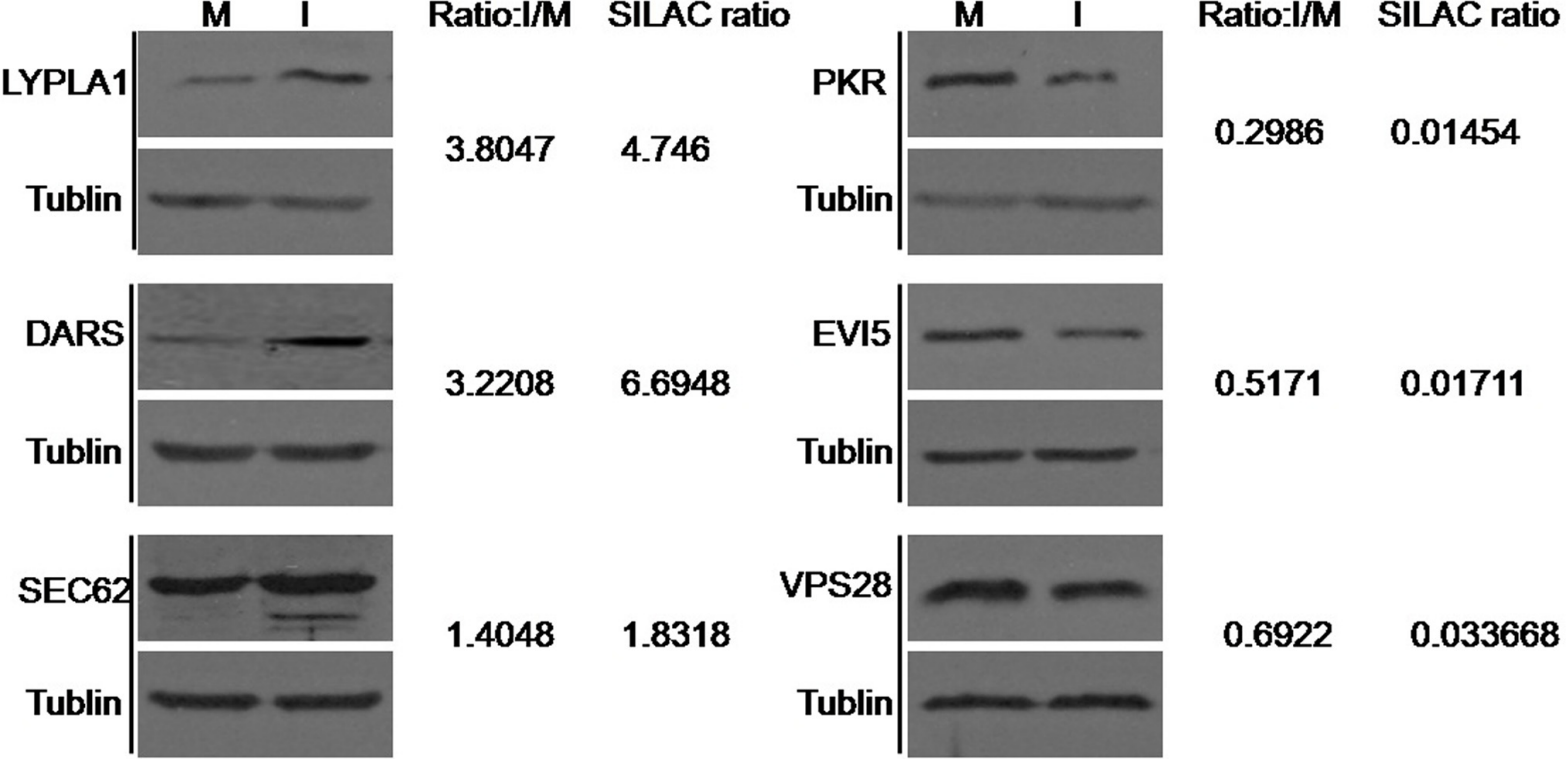
Confirmation of six up-regulated or down-regulated proteins by immunoblot compared to the SILAC analysis. Relative expression of six representative proteins (normalized to actin band) was determined using image densitometry by software Image J. The ratio of I/M is reflected by the band densitometry of infection compared to that of mock infection. M, Mock; I, infected.

By importing the protein GI numbers and regulation levels into the Ingenuity Pathway Analysis (IPA) tool, the interacting pathways were constructed. A total of 14 pathways were identified at 95% or greater confidence level. Among them, 11 pathways have involved 11 or more significantly up- or down-regulated proteins (the so called “focus” members) (S4 Fig, S2 and S3 Tables). As suggested by network analysis, it appears that most proteins with altered abundance are involved in cellular assembly, organization, morphology, signaling and growth in FMDV-infected cells. For example, many of the proteins with significant regulation were linked to EIF2AK2 (also named PKR, double-stranded RNA-dependent protein kinase R) signaling network. Thus, combined with the significance of regulation, the five networks of interest are as follows: (1) Protein Synthesis, Gene Expression, Nucleic Acid Metabolism (Fig 4A); (2) Cell Death, Inflammatory Response, Infectious Disease (Fig 4B); (3) Hematological System Development and Function, Hematopoiesis, Cellular Development (Fig 4C); (4) Gene Expression, Cell Death, Cell Morphology (Fig 4D); (5) DNA Replication, Recombination and Repair, Cancer, Skeletal and Muscular Disorders (Fig 4E). Among these networks, up-regulated DARS and SEC62 proteins and down-regulated VPS28 and EVI5 significantly were involved in the PKR pathway. Only LYPLA1 protein bore no relation to PKR, but also was up-regulated significantly. As shown in Fig 4, identified up-regulated proteins present in the pathways are depicted in shades of red; proteins present in the pathways and identified as down-regulated are shown in green; and proteins present in the pathways and identified with no change in regulation are depicted in white. The intensity of color indicates the level of regulation.

### Validation of SILAC ratios by western blot

Combined with the results of IPA and calculation of MaxQuant, SEC62 and DARS in FMDV-infected cells are up-regulated significantly, whereasVPS28 and EVI5are down-regulated significantly. Although the LYPLA1 protein is not in the network related to EIF2AK2, its expression was also up-regulated significantly (ratio = 4.746). Thus, to rigorously validate the identification and the SILAC-determined protein ratios, we analyzed six selected proteins, including the up-regulated (SEC62, DARS, and LYPLA1) and the down-regulated (EIF2AK2, VPS28, and EVI5) proteins in infected and mock-infected cells by immunoblotting. As shown in Fig 5, the Western blot analysis results showed the consistency of the ratios of the six representative proteins in infected and uninfected cells with those obtained from SILAC.

### Potential relocalization of cellular proteins in FMDV-infected cells

**Fig 6:**
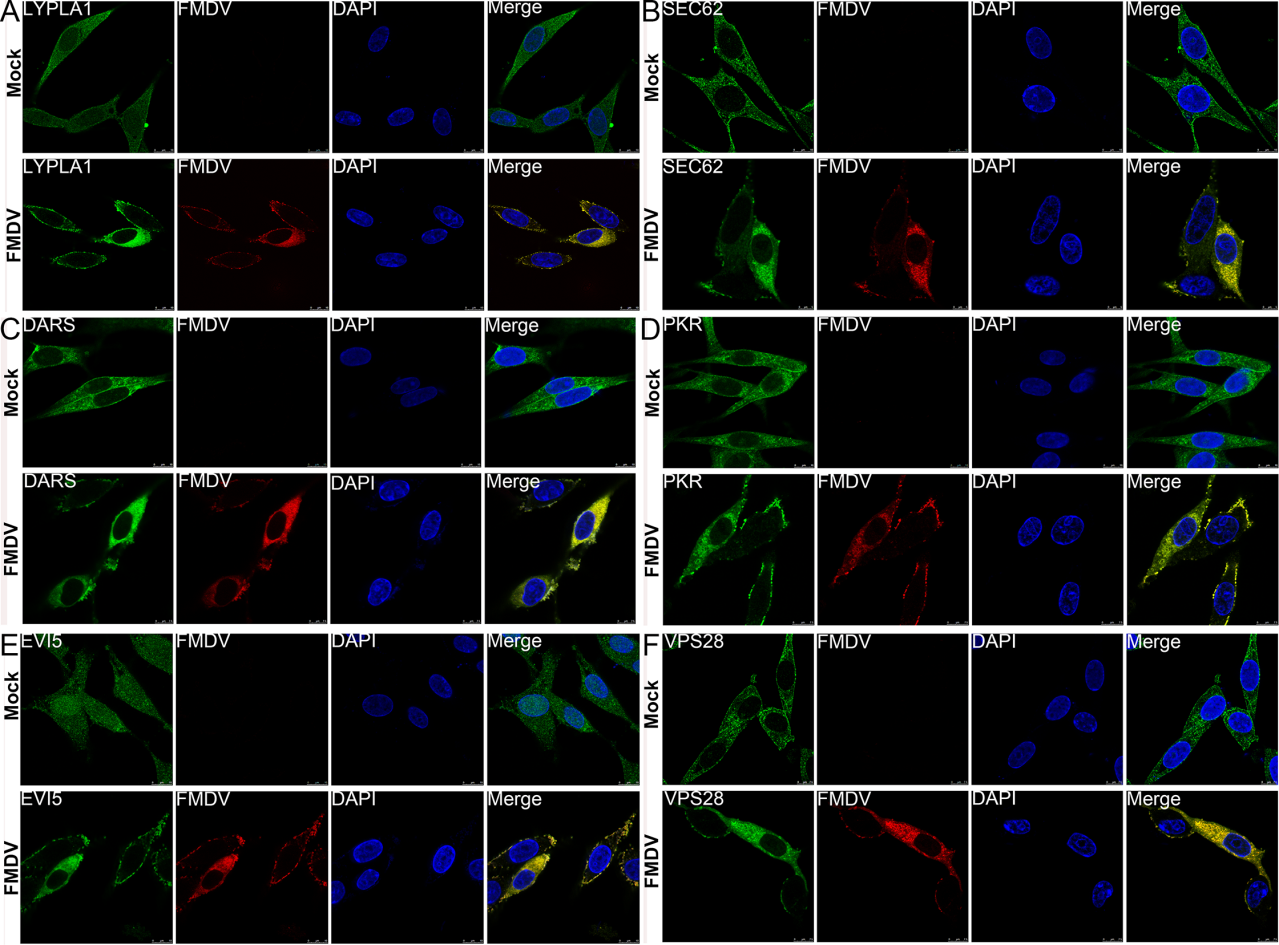
Subcellular localization of target proteins in mock- and FMDV-infected BHK-21 cells by confocal microscopy analysis. Target proteins are stained green, FMDV proteins are shown in red, and the nuclei are stained blue with DAPI.

To investigate whether the six cellular proteins were redistributed in FMDV-infected cells or co-localized with FMDV capsid protein, BHK-21 cells were infected with FMDV, and their subcellular localization was determined at6 h.p.i by using specific antibody against six cellular proteins, respectively. As shown in Fig 6, except for the EVI5 protein which is localized to both the cytoplasm and the nucleus in mock-infected cells, other proteins including SEC62, DARS, PKR, LYPLA1 and VPS28 were localized in the cytoplasm. Upon FMDV infection, all six proteins were co-localized with the FMDV capsid proteins either in the cytoplasm or in the peripheral cytoplasm (Fig 6). EVI5 mainly re-localized with the FMDV capsid in the cytoplasm, with no remaining detection in the nucleus (Fig 6E). The translocation and colocalization of the six host proteins with the major FMDV proteins suggest that these host cell proteins may play a functional role in FMDV replication.

### Potential importance of cellular proteins in the FMDV life cycle

**Fig 7:**
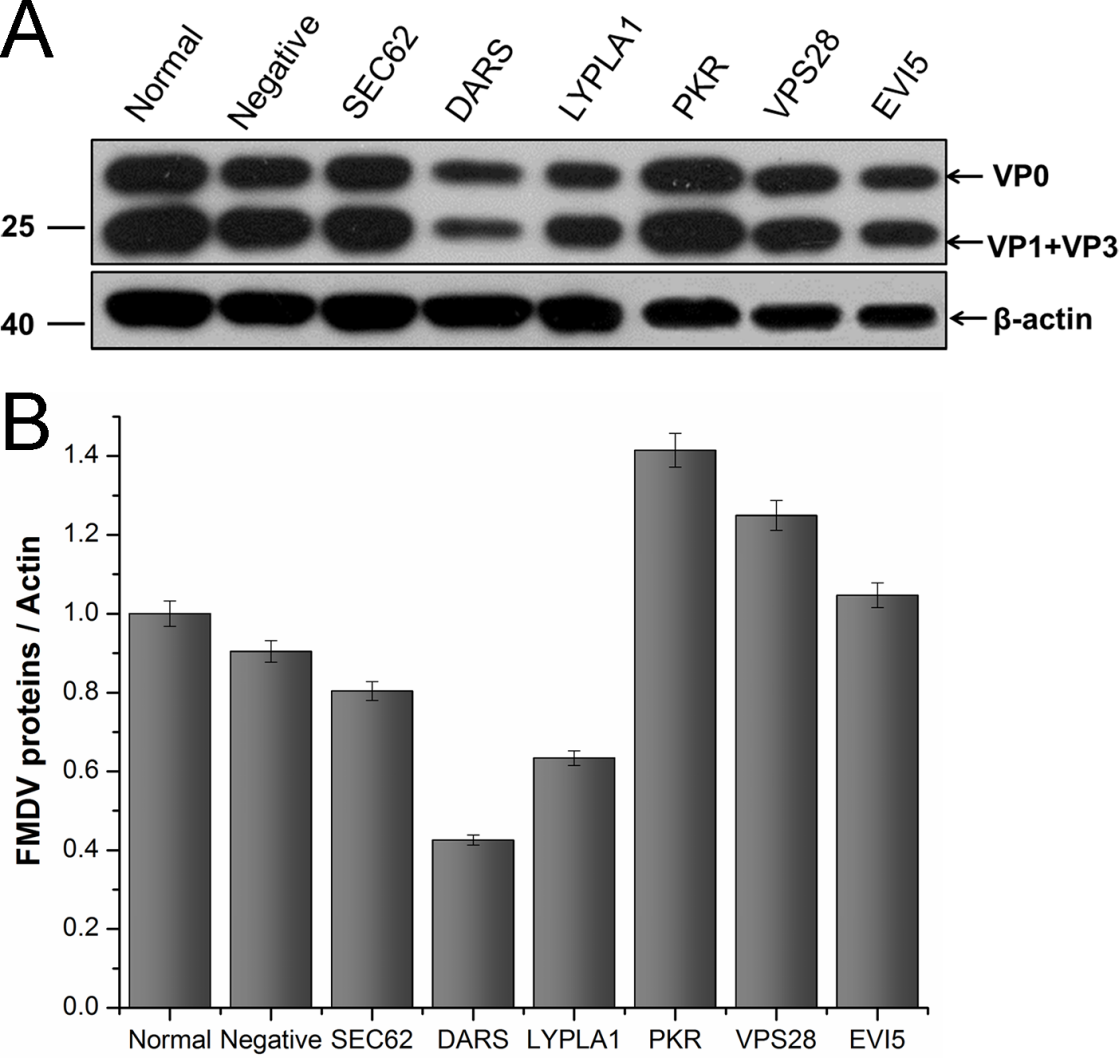
Effects of representative protein on FMDV replication in BHK-21 cells were confirmed by western blot analysis after RNA interference. FMDV capsid protein was detected by the pig anti-FMDV Asia 1 serum. Relative expression of FMDV proteins was determined using image densitometry by software Image J. The relative quantity of FMDV protein was showed by normalization of ratio of FMDV capsid protein to actin with that ratio of normal cells. Bars represented the means±standard deviations (SD) of three independent experiments.

**Fig 8:**
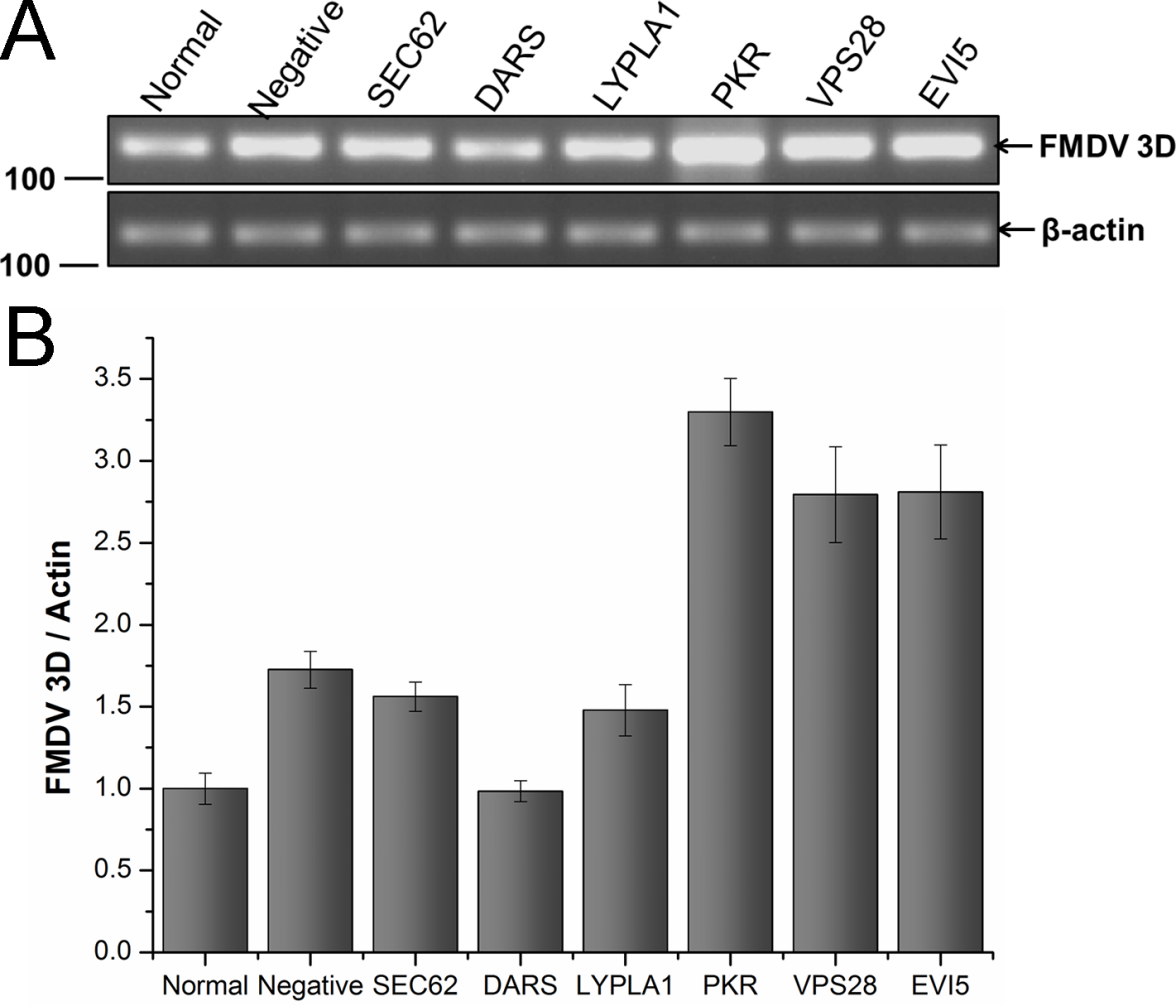
Quantification of FMDV gene in BHK-21 cells after knockdown genes of representative proteins. The cells transfected with untargeting siRNA was set as negative control, the cells untransfected was blank control. FMDV RNA was indicated by the FMDV 3D gene which is conservative and stable in the FMDV genome. Relative quantity of gene was determined using image densitometry by software Image J. The relative quantity of FMDV gene was showed by normalization of ratio of FMDV 3D gene to actin gene with that ratio of normal cells. Bars represented the means±SD of three independent experiments.

**Fig 9:**
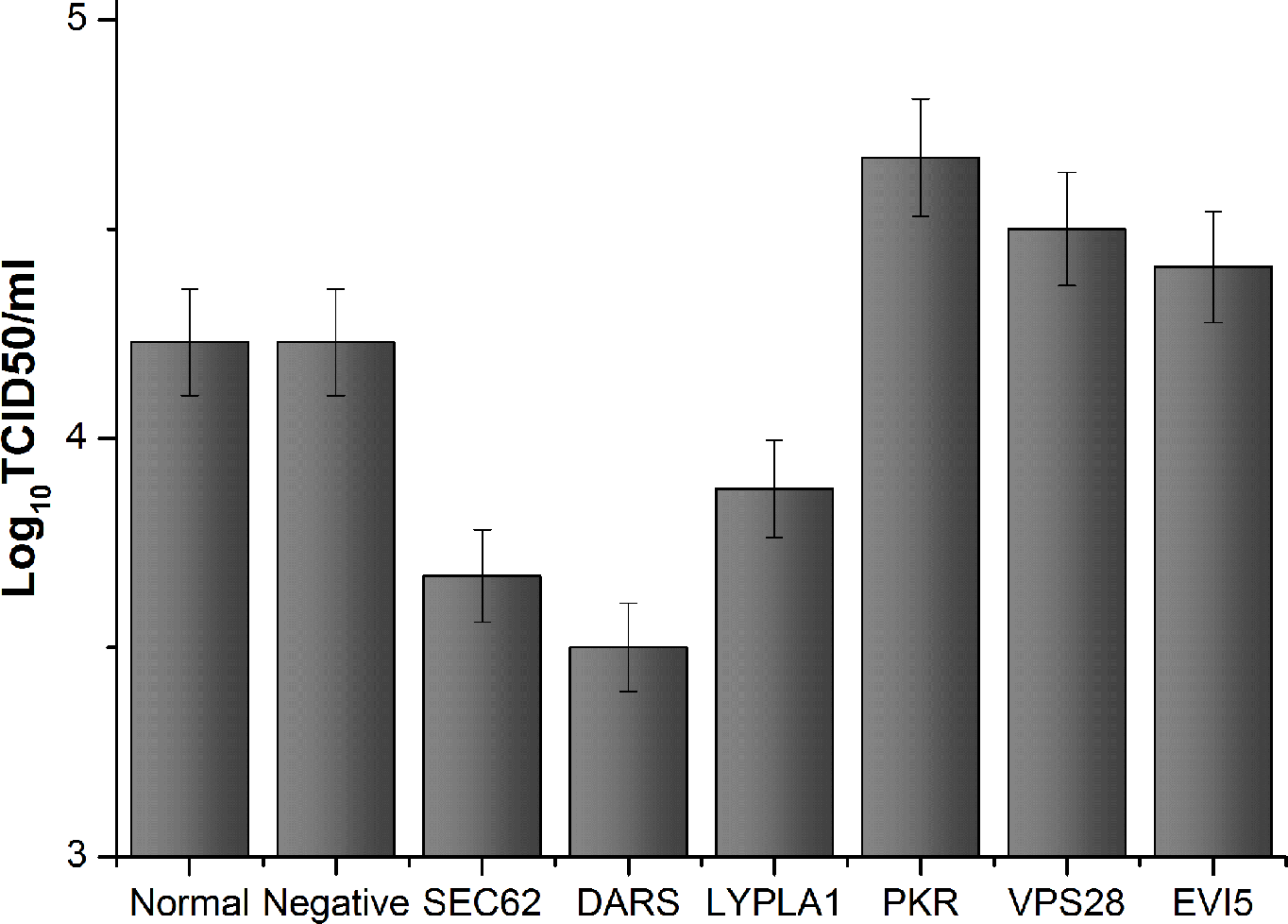
FMDV titer was detected by the TCID50 after transfection with siRNA or untargeting siRNA. The cells transfected with untargeting siRNA was set as negative control, the cells untransfected was blank control. Bars represent the means±SD of three independent experiments.

To investigate the importance of these cellular proteins in the FMDV life cycle, BHK-21 cells were transfected with specific siRNAs for each mRNA coding for the selected cellular target proteins. Cell pellets were collected at 30 h after siRNA transfection and analyzed by western blotting. The results indicated that the expression of all six proteins was decreased in the presence of specific siRNA, but not with the non-targeting siRNA (S5 Fig) at 30 h after transfection when compared to cells untreated with siRNA. In the subsequent experiments, the time point of 30 h post-siRNA transfection was chosen. At this time point, the transfected cells were infected with FMDV at an MOI of 1.To identify the functions of the six target proteins in FMDV infection, cells and cell culture medium were collected for western blot, RT–PCR, and TCID_50_ assay at 6 h after FMDV infection. Western blot analysis indicated that the abundance of FMDV capsid protein in SEC62-, DARS-, or LYPLA1-knockdown cells decreased compared with that of the negative control (nontargeting siRNA-treated cells) or normal cell control (untreated cells) (Fig 7). Among these cells, DARS-knockdown cells induced a significant decrease of FMDV capsid (Fig 7). However, in PKR-, VPS28-, or EVI5-knockdown cells, the abundance of FMDV capsid proteins increased, compared with that of the negative control (nontargeting siRNA-treated cells) or normal cell control (untreated cells) (Fig 7), which suggested the target proteins could affect the translation or expression of FMDV protein in cells. To identify whether the target proteins impact the replication of FMDV at the genome level, RT–PCR was used to investigate the abundance of FMDV 3D gene under different conditions using a specific primer pair (Fig 8). The data indicated that the amount of genomic RNA decreased in infected cells reduced of SEC62, DARS, or LYPLA1, but the amount of genomic RNA increased in infected cells depleted of PKR, VPS28, or EVI5 (Fig 8). It demonstrated that these host proteins could play a role during the replication of FMDV. To further study whether the target proteins may affect the assembly or release of FMDV, the FMDV titer in the cell culture medium was evaluated after the siRNA-mediated knockdown of these proteins (Fig 9). Variations in the FMDV titer were consistent with the viral protein and RNA levels, confirming that these host proteins may affect FMDV replication.

## Discussion

A comprehensive knowledge of host cell protein expression profiles during FMDV infection would provide significant insights into the molecular mechanisms that regulate viral replication and pathogenesis. This may lead to the identification of cellular targets for the development of a new generation of antiviral drugs against essential host cell factors. This study aims to provide an unbiased global overview of the protein expression profiles in cells infected with FMDV through quantitative proteomics and subsequent bioinformatics analysis using IPA. The study highlights new interactions between FMDV and cellular proteome in BHK-21 cells *in vitro*. As we know, there are seven serotypes of FMDV which have different biological characteristics in infection of animals, suggesting that it would be necessary to comparatively study the interaction of each FMDV serotype with the host cell. Extending these types of analyses to other cell types and FMDV serotypes could lead to the identification of common and unique features in host interaction with individual serotypes and may provide an approach for ideal antiviral therapies. As far as we were aware, this study may represent the first quantitative proteomic analysis of cells infected with FMDV serotype Asia 1.The reason for selection of FMDV serotype Asia 1/Jiangsu/China/2005 as a model in this study is due to the fact that this strain is widely used as a vaccine strain in China and its biological property is well defined. The quantitative proteomic analysis on serotype Asia 1/Jiangsu/China/2005 would be the most representative for other FMDV serotype Asia 1 which is endemic in the Asia. In addition, BHK-21 cell line was selected as model because it is commonly used in investigating FMDV host–pathogen interactions, antiviral screening and vaccine production. Although the hamster genome database has poorer annotation compared with the mouse genome and many proteins are unassigned or uncharacterized, the origin of BHK-21 cell is hamster, which belongs to rodent species and has close relationship with mice. Hence, gene identifications of the identified proteins can be converted to mouse protein GI numbers, which can minimize the possibility of information missing error. It showed a majority of the cellular proteome remained relatively unchanged in virus-infected BHK-21 cells, suggesting that only specific changes occurred in response to virus infection. Proteins were identified and quantified at 6 h post-infection, prior to the appearance of major CPE. At this time point, the majority of the infected cells would remain viable for downstream processing. In this study, network pathway analysis indicated that the cellular proteins in the proteomes could be linked to signaling cascades, such as PKR. Interestingly, several significant up- and down-regulated proteins, including DARs, SEC62, VPS28 and EVI5, were related to PKR in different networks. Hence, in this study, DARs, SEC62, VPS28, EVI5 and PKR were selected to verify their functional importance in FMDV infection. Although, LYPLA1 seems to have no relationship with PKR, its expression increased significantly in BHK-21 cells infected with FMDV. Hence, LYPLA1 was also studied in this report.

PKR is a serine and threonine protein kinase that was expressed ubiquitously and identified as an antiviral and antitumor protein induced by interferon (IFN)[17]. This kinase contributes to inflammation and immune regulation through several signaling pathways and can be activated by multiple stimuli [17, 18]. Active PKR can mediate the activation of mitogen-activated protein kinases [19, 20], inhibitor of κB kinase [21, 22], and IFN-β-promoter simulator 1 (IPS-1) signaling [23]. Thus, PKR has multifaceted functions in the regulation of inflammatory and immune responses. As for the relationship of PKR and FMDV, previous research showed an important function of PKR in blocking FMDV replication at the protein translation level. In support of these findings, porcine and bovine cells treated with 2-aminopurine, an inhibitor of PKR, increased the virus yield for several folds compared with that of the untreated infected cells [24]. In addition, immediate-early induction of IFN-mRNA and mRNAs for some IFN-stimulated gene products, such as 2′5-oligoadenylate synthetase, Mx1 and PKR could be reduced by FMDV L^pro^ protein in swine cells. Knockdown of cellular PKR by RNA interference did not show obvious effect on wild-type virus yield, but resulted in a higher yield of A12-LLV2, a mutant virus lacking the L^pro^ protein. These results indicated a direct function of PKR to control FMDV replication in the natural host and the relationship of FMDV L^pro^ and PKR[25]. Thus, PKR was selected as a positive control in our study. In quantitative proteomics, the expression of PKR decreased in cells infected with FMDV, suggesting that there is an antagonism between PKR and FMDV. Consistent with this speculation, cellular PKR down-regulation by RNA interference resulted in a higher titer of FMDV, which further confirmed a direct function of PKR in controlling FMDV replication combined with previous research. Therefore, previously published data were also reflected by the bioinformatics analysis of the current quantitative proteomics data, and on the other hand indicated that the results of RNA interference assay in our study were reliable and positive.

The endoplasmic reticulum (ER) is the cellular site for production of secretory proteins, lipids, cell membranes and sterol. With the aid of molecular chaperons, newly synthesized proteins are folded and glycosylated in the ER lumen before budding and transport to the Golgi apparatus. A number of cellular stress conditions may interfere with the ER function and cause the accumulation of misfolded and unfolded proteins in the ER, leading to the initiation of a group of signal transduction pathways, known as the ER stress response or unfolded protein response [26, 27]. Interestingly, ER is also a cellular organelle exploited by many viruses for replication and maturation [28, 29]. In virus-infected cells, ER stress response can be induced, either by overwhelming the protein folding capability or by directly disrupting the ER structure and function, to facilitate viral replication and pathogenesis in some cases [30, 31]. Certain nonstructural proteins, such as 2B, 2C and 3A from some picornaviruses, are implicated in disrupting the ER integrity and functions [32–34]. On the other hand, some viruses in the picornavirus family may require ER membranes for the biogenesis of viral proteins and for anchoring the replication complexes [34–36]. In the present study, SEC62 was up-regulated after FMDV infection, suggesting that SEC62 proteins are beneficial to FMDV proliferation. This finding is consistent with the decrease of FMDV replication after knockdown of SEC62 protein expression.Sec62 has been found to interact with the ribosome, which suggests its function in co-translational translocation in mammalian cells [37]. Sec62 and Sec63 are important components that act in post-translational translocation at the mammalian ER for precursor chain of proteins [38]. Thus, SEC62–SEC63 balance is expected to contribute to the development of human tumorigenesis, possibly by relocation of the uncomplexed SEC62 to a yet undetermined cellular location along the secretory pathway. However, the association of the overexpression of SEC62 after FMDV infection with ER stress, as well as the mechanisms of ER stress that affect FMDV proliferation, is not clearly elucidated. The clarification of this mechanism, especially, the relationship between ER stress and FMDV replication, is beneficial in understanding the FMDV pathogenesis.

The basic function of aminoacyl-tRNA synthetases (ARSs) is to ensure the accurate transfer of information directed by the genetic code and ensure the correct expression of proteins[39]. During evolution, certain ARSs also acquire appended domains responsible for a range of noncanonical activities unrelated to aminoacylation[40]. These noncanonical activities include translation control, transcription regulation, cell migration, signal transduction, inflammation, angiogenesis, and tumorigenesis. Defects in both canonical and noncanonical ARS functions may cause or contribute to human disease [41].DARS belongs to class II ARSs and exists as a dimer. The pathophysiology of the disorder, including slowly progressive cerebellar ataxia, spasticity and dorsal column dysfunction, is elucidated with the discovery of mutations in the DARS-encoding gene [42, 43]. However, no reports are available on DARS in virus-infected cells. DARS is up-regulated after FMDV infection in BHK-21 cells by SILAC. This finding suggests that DARS could be useful for FMDV proliferation, especially, for FMDV protein translation, as confirmed by the RNA interference result of the DARS-encoding gene. Interestingly, DARS gene is the first known calcium-regulated tRNA synthetase in *Dictyosteliumdiscoideum*, suggesting a novel mechanism by calcium to regulate the translation machinery [44]. Combined with previous research, the gradual enhancement of membrane permeability because of the formation of membrane-embedded pores of picornovirus 2B protein, which disrupts intracellular Ca^2+^ homeostasis, is shown [45–48]. The increase in cellular Ca^2+^ after FMDV infection could promote the expression of DARS gene, which is consistent with the result of SILAC and RNA interference. This finding would warrant further studies of the relationship between DARS and the expression of 2B protein as well as the 2B-mediated regulation of Ca^2+^concentration during FMDV infection.

Protein acylation, including isoprenylation (farnesylation and geranylgeranylation), glycosyl phosphatidylinositol anchoring, myristoylation and palmitoylation, refers to the covalent addition of a fatty acid prosthetic group onto an acceptor protein. Among these modifications, myristoylation and palmitoylation are the two major types of acylmodification. Protein acylation would provide hydrophobicity for proteins to be localized at the membrane and may facilitate protein–protein interactions [49, 50]. Several viral capsid or envelope proteins have been found to be modified bymyristoylation and palmitoylation. This may enhance the hydrophobic characteristics of viral proteins for their correct subcellular localization as well as to facilitate the assembly and disassembly of infectious virions[51]. A number of protein acyltransferase and acylproteinthioesterase (APT or LYPLA1) catalyze the palmitoylation and depalmitoylation processes. Among them, APT1 is the enzyme responsible for cleavage of the palmitoyl group[52]. As previously noted, most viral acyl proteins are capsid or envelope constituents involved in capsid assembly or in the entry of viruses into the cells. We speculated that palmitoylation and depalmitoylation processes are involved in virion assembly and production of infectious progeny. LYPLA1 expression was up-regulated after FMDV infection and the virus replication decreased after knockdown of LYPLA1 expression, which demonstrated that LYPLA1 could improve FMDV replication, especially for assembly and production of infectious progeny. However, the detailed mechanism in which LYPLA1 modifies the FMDV capsid proteins should be further studied.

Endosomal sorting complex required for transport (ESCRT) complexes and associated protein play a function in membrane fission events, such as multi vesicular body (MVB) formation and terminal stages of cytokinesis [53, 54]. The ESCRT machinery is also required for the budding of numerous enveloped viruses to cut the membranous neck that connects the virion to the plasma membrane [55]. This machinery includes ESCRT-I, ESCRT-II, and ESCRT-III, as well as other associated proteins, such as ATPase vacuolar protein sorting 4 (VPS4). ESCRT-I proteins (such as tumor susceptibility gene 101, Tsg101) and associated proteins (Alix) have function at the early stage in this pathway, but ESCRT-III proteins (charged MVB proteins, Chmp) have function in the late stage and are disassembled by VPS4 [56]. Previous research has also demonstrated that mammalian TSG101 binds VPS28 directly to form ESCRT-I and TSG101. Its interacting components are directly involved in endosomal sorting, which suggests that VPS28 could have function in virus infection [57]. Although limited information on non-enveloped viruses is available, previous studies have shown that α2β1 integrin clustering triggers α2-MVBs. This phenomenon depends on ESCRT complexes that seem to crucial for promoting non-enveloped picornavirus echovirus 1 infection [58]. Hepatitis A virus (HAV) released from cells is wrapped in host-derived membranes, which may facilitate virus escape from neutralizing antibodies and promote virus spread within the liver. These enveloped viruses (“eHAV”) resemble exosomes, which are small vesicles whose biogenesis is dependent on host proteins associated with ESCRT, VPS4B, and ALIX. Interestingly, in this study, we showed that FMDV infection down-regulated VPS28 protein expression and the virus titer increased after knockdown of the VPS28 gene. This finding suggests that the VPS28 protein may function as a suppressor in FMDV infection, which is inconsistent with a previous study [58]. Further studies would be required to clarify the relationship between FMDV and VPS28.

EVI5 is a Rab11-binding protein that acts as a GAP protein for Rab11. Rab11 regulates intracellular transport and has specific functions in endosome recycling and cytokinesis [59, 60].Many studies have shown that Rab11 involved in cellular recycling endosome pathway and has a function in enveloped viral budding and release, such as respiratory syncytial virus [61, 62], influenza A virus [63], Andes virus [64] and Sendai virus [65], suggesting a possible function for the Rab11 pathway akin to that of the ESCRT machinery. A previous study has demonstrated that FMDV infection occurs predominantly from within early endosome and does not require virus trafficking to the late endosomal compartments. Expression of dominant-negative Rab11 mutants inhibited infection by FMDV up to 35% [66], which indicates that Rab11 also functions during FMDV infection. However, the present study showed that EVI5, a rab11-binding protein, was down-regulated after FMDV infection. FMDV titer increased after knockdown of EVI5 expression. The results suggested that EVI5 protein could inhibit FMDV infection, which further implies the opposite function of Rab11 and EVI5 for FMDV infection. It would be interesting to elucidating the relationship of Rab11 and EVI5 in FMDV infection.

During viral replication cycle, the genome is rapidly translated into polyprotein upon FMDV entry into a cell. The functional capacity of the polyprotein involved in viral replication, protein translation and protein modification in the host cells, will increase when cellular changes occur during viral replication, and will further affect the cellular pathways, which are critical for viruses [67]. It suggests that the virus proteins could also affect cellular response during virus proliferation in cells. Thus, determining the relationship of certain virus proteins through cellular interactome is also necessary in the future.

## Conclusions

In this study, we have applied SILAC to study the regulation of 2,141 cellular proteins in BHK-21 cells infected with FMDV serotype Asia 1. Most proteins measured by this nonbiased approach were not altered significantly with L/H ratios around 1.0, confirming that no attempt to enrich for any subpopulation of proteins or specific modifications was made experimentally. The results showed that the abundance and expression patterns of 153 proteins were significantly changed upon FMDV infection. Network pathway analysis indicated that cellular proteins with significant regulation could be linked to signaling cascades, such as PKR. Interestingly, several significant up- and down-regulated proteins, including DARs, SEC62, VPS28 and EVI5, were related to PKR in different signaling networks. Furthermore, DARs, SEC62, VPS28, EVI5, PKR and LYPLA1 were selected and validated. The results suggest that FMDV infection may have effects on the expression of specific cellular proteins to create more favorable conditions for FMDV infection. This study may lead to the identification of cellular targets for the development of a new generation of antiviral drugs against essential host cell factors.

## Supporting Information

S1 Fig The schematic procedure of SILAC. (PDF)

S2 Fig The analysis of the incorporation of lysine (13C6 HCl-Lys) by MALDI-TOF. (PDF)

S3 Fig GO analysis. (PDF)

S4 Fig Overview of 11 specific functional networks, each containing 11 or more “focus” proteins (proteins that were significantly up-regulated or down-regulated). (PDF)

S5 Fig Efficiency of siRNA knockdown. (PDF)

S1 Table The overall analysis of protein variance. (XLSX)

S2 Table Network summary. (DOCX)

S3 Table Details of 11 Networks. (DOCX)
