# Supplementary material for: Quantitative Proteomic Analysis of BHK-21 Cells Infected with Foot-and-Mouth Disease Virus Serotype Asia 1
Source: PLoS One. 2015 Jul 10;10(7):e0132384. doi: 10.1371/journal.pone.0132384 (PMC4498813; doi:10.1371/journal.pone.0132384)
Supplement: S1 Fig — (PDF) [file pone.0132384.s001.pdf]

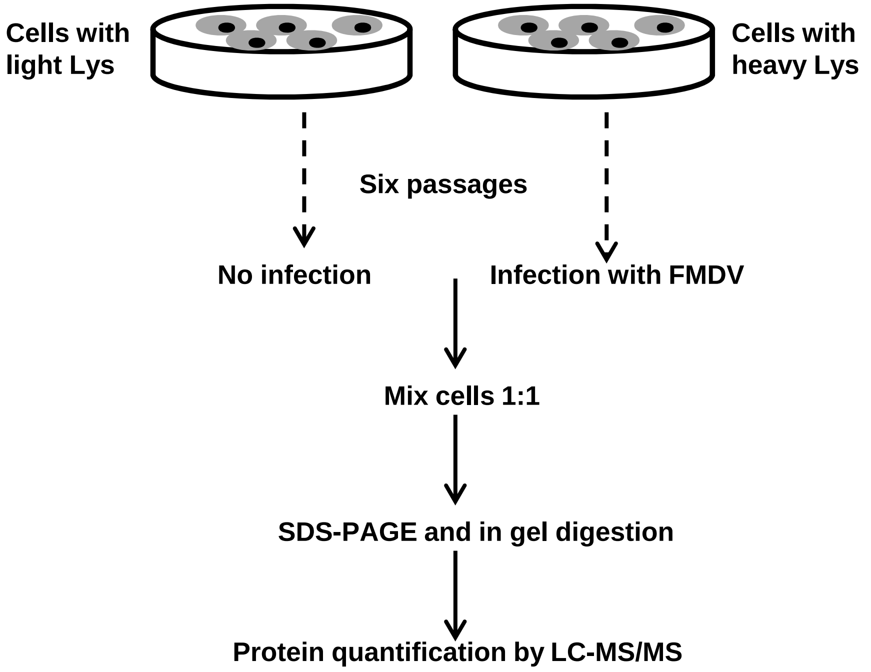

S1 Fig. The schematic procedure of SILAC. Cells were grown in either the heavy or the light culture medium for six passages at least after which the incorporation of the heavy forms of lysine (13C6HCl- Lys) was analyzed by MALDI-TOF MS
