# Supplementary material for: Quantitative Proteomic Analysis of BHK-21 Cells Infected with Foot-and-Mouth Disease Virus Serotype Asia 1
Source: PLoS One. 2015 Jul 10;10(7):e0132384. doi: 10.1371/journal.pone.0132384 (PMC4498813; doi:10.1371/journal.pone.0132384)
Supplement: S2 Fig — (PDF) [file pone.0132384.s002.pdf]

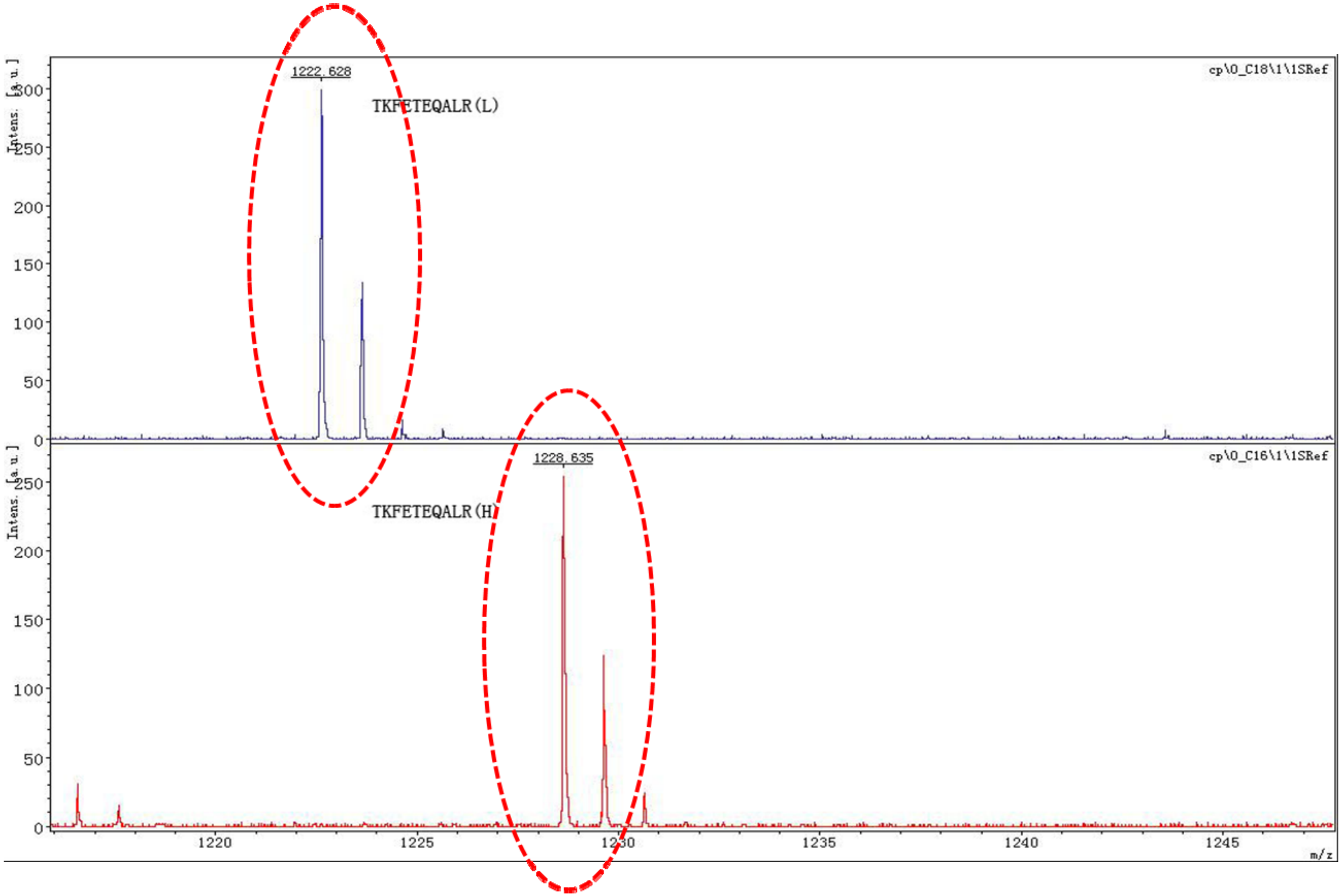

S2 Fig. The analysis of the incorporation of lysine (13C6 HCl-Lys) by MALDI-TOF. Two randomly chosen peptides from cells were analyzed. Peaks in these mass spectra correspond to unlabeled peptides with a mass of 1222 and 1008 Da, respectively. The lower panels show the same peptides from cells that had been passaged six times in the heavy medium, respectively. The peaks show a mass difference of 6Da (1008 versus 1014, 1B, 1222 versus 1228), corresponding to the incorporation of a heavy 13C6HCl-Lys.
