# Supplementary material for: Quantitative Proteomic Analysis of BHK-21 Cells Infected with Foot-and-Mouth Disease Virus Serotype Asia 1
Source: PLoS One. 2015 Jul 10;10(7):e0132384. doi: 10.1371/journal.pone.0132384 (PMC4498813; doi:10.1371/journal.pone.0132384)
Supplement: S3 Fig — (PDF) [file pone.0132384.s003.pdf]

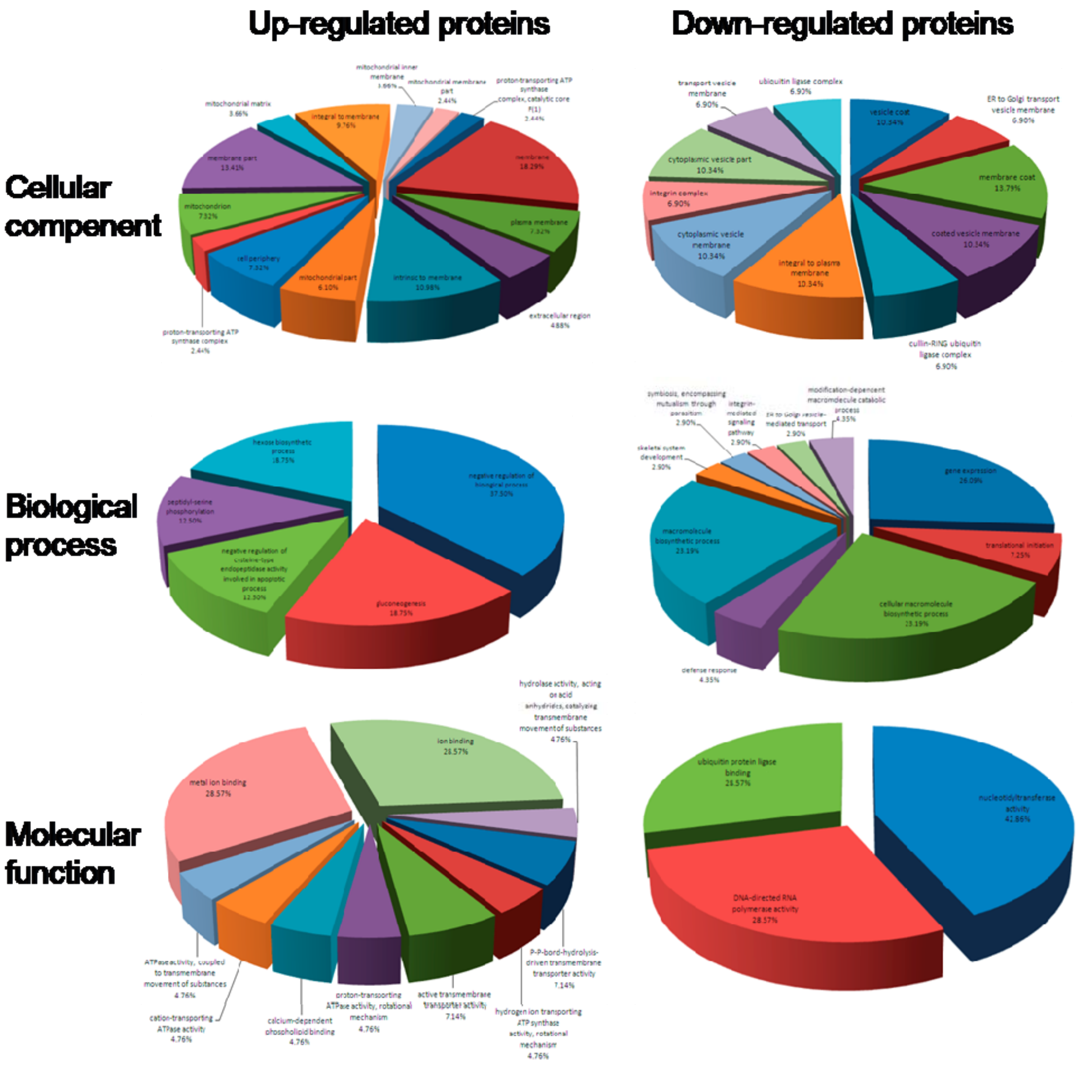

S3 Fig. GO analysis. To gain functional insights into the cellular proteome, the 163 identified proteins were assigned to different molecular functional classes and subcellular annotations based on the underlying biology evidence from the UniProtKB/Swiss-Prot and TrEMBL protein databases and the Gene Ontology database. Because the hamster genome database had poor annotation compared to the mouse genome and many proteins were unassigned or uncharacterized, gene identifications of the identified proteins in S1 Table were converted to mouse protein gi numbers. Protein gi numbers and levels of regulation were imported into the Ingenuity Pathways Analysis (IPA) tool, and interacting pathways were constructed based on the underlying biological evidence from the literature database. The total identified proteins in infected cells were classed into different molecular and cellular functional groups, including Biological Process (BP), Cellular Component (CC), Molecular Function (MF).
