# Supplementary material for: Quantitative Proteomic Analysis of BHK-21 Cells Infected with Foot-and-Mouth Disease Virus Serotype Asia 1
Source: PLoS One. 2015 Jul 10;10(7):e0132384. doi: 10.1371/journal.pone.0132384 (PMC4498813; doi:10.1371/journal.pone.0132384)
Supplement: S4 Fig — (PDF) [file pone.0132384.s004.pdf]

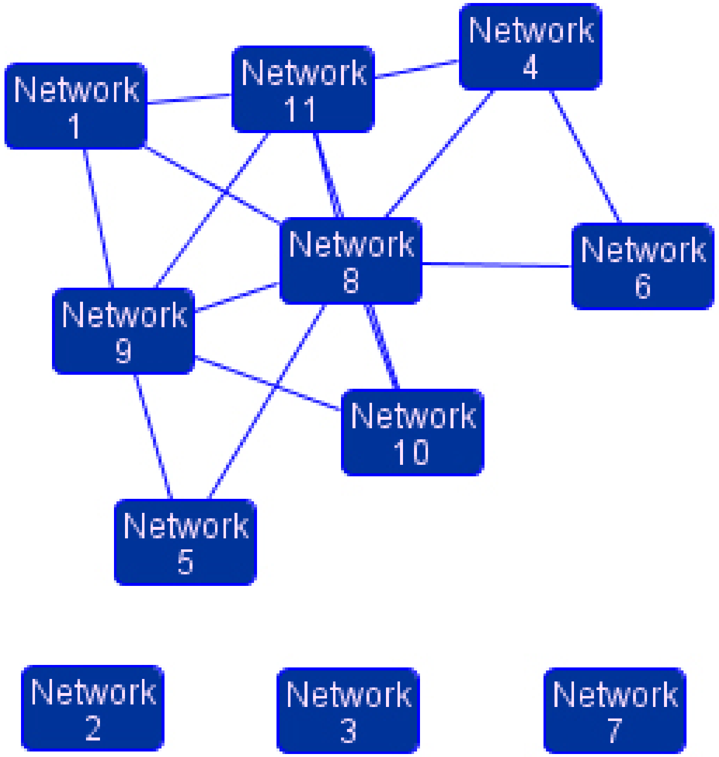

S4 Fig. Overview of 11 specific functional networks, each containing 11 or more "focus" proteins (proteins that were significantly up-regulated or down-regulated). Each box contains an arbitrary network number. The line between the two networks represents the presence of overlapped proteins. More information is available in Supplemental material S2 and S3 Tables.
