# Supplementary material for: Quantitative Proteomic Analysis of BHK-21 Cells Infected with Foot-and-Mouth Disease Virus Serotype Asia 1
Source: PLoS One. 2015 Jul 10;10(7):e0132384. doi: 10.1371/journal.pone.0132384 (PMC4498813; doi:10.1371/journal.pone.0132384)
Supplement: S5 Fig — (PDF) [file pone.0132384.s005.pdf]

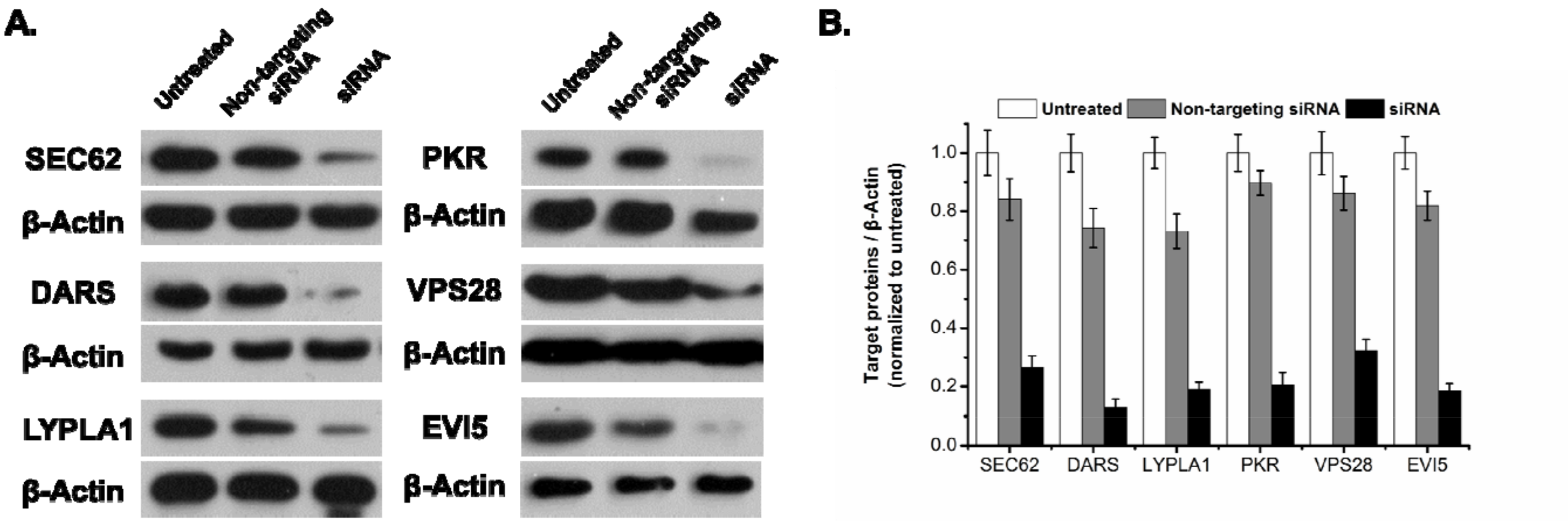

S5 Fig. Efficiency of siRNA knockdown. To validate the functional knockdown of the targeted genes, protein expression level of each gene were determined after siRNA transfection by specific antibody. The relative expression of representative proteins was evaluated by the ratio of target protein to actin. Control means BHK-21 cells transfected with non-specific siRNA control. siRNA transfection means BHK-21 cells tranfected with specific siRNA against gene of representative protein.
