# Supplementary material for: Quantitative Proteomic Analysis of BHK-21 Cells Infected with Foot-and-Mouth Disease Virus Serotype Asia 1
Source: PLoS One. 2015 Jul 10;10(7):e0132384. doi: 10.1371/journal.pone.0132384 (PMC4498813; doi:10.1371/journal.pone.0132384)
Supplement: S2 Table — (DOCX) [file pone.0132384.s007.docx]

**S2 Table. Network summary.**

| **ID** | **Molecules in Network** | **Score** | **Focus**  **Molecules** | **Top Functions** |
| --- | --- | --- | --- | --- |
| 1 | Akt,ATP5A1,ATP5D,ATPAF2,CDC5L,CHTOP,DARS,EIF1,Eif2,EIF1B,EIF2AK2,EIF2S3,EIF4A3,GPI,MTORC1,RBM7,Ribosomal 40s subunit, Rnr, RPS3, RPS6, RPS12, RPS24, RPS26,RPS3A,SARNP,Scd3,Scd4,SCD,Spectrin,SPTB,SPTBN1,SPTBN2,SRRM2,stearoyl-CoA 9-desaturase,UPF1 | 57 | 28 | Protein Synthesis, Gene Expression, Nucleic Acid Metabolism |
| 2 | Actin,ARPC2,ATPase,BTF3,Calmodulin,CD44 (includes EG:100330801),Ck2,EDF1,ERK,Estrogen Receptor, FActin, Holo RNA polymerase II,Hsp90, MYH1, MYH2, MYH3, MYH4, MYH6,MYH7, MYH8,Myosin, MYRIP, NEDD4, PDCD6IP, PLS1,POLR2C,POLR2G,RNA polymerase II, SAFB2, SAFB,SUGT1, TCEA1,TIMM17B, TOMM70A, UBAP2L | 46 | 24 | Cell Morphology, Infectious Disease, Skeletal and Muscular System Development and Function |
| 3 | Alp,ANXA5,C1QBP,CCDC6,collagen,Collagen Alpha1, Collagen type I, Collagen type III,Collagen type IV, Collagen(s),COPA,CTSL2,DNAJA3,EIF4G2,Eotaxin,ERK1/2,Fcer1,GBAS,ITGB3,Laminin1,LGALS3,Mlc,MRC2,MYL4,p70 S6k,Pdgf (complex),PDGF BB,PLC gamma,PP2A, PRKAA1, RPL23A,RPTOR,SLC25A11,Tgf beta,TIMP1 | 27 | 17 | Cellular Growth and Proliferation, Connective Tissue Development and Function, Hepatic System Development and Function |
| 4 | C11orf58,CAPRIN1,CHCHD6,CPSF7,CRIPT,CUL5,DAD1 (includes EG:13135),DLG4,DLST, DNAJC11,FXYD7, G3BP1, GPAA1,HNF4A,IFI30,KRI1, MTMR2,MTX1,MTX2, NDFIP1,NEDD4L,NOC3L,OGFOD1,PGM3,PGRMC2,PIGS,SAMM50,SLIRP,SSSCA1,TGFB1 (includes EG:21803), TOE1, UMPS,WDR36,YKT6,ZNF207 | 25 | 15 | Cellular Compromise, Cell-To-Cell Signaling and Interaction, Skeletal and Muscular System Development and Function |
| 5 | BCR,BSG (includes EG:12215),Caspase,CUL3 (includes EG:26554),CyclinA,CyclinE,Cytochrome c,DDX5, DDX17, DDX3X, E2f,HRAS,IFN Beta,Ifn gamma,Igm, Immunoglobulin, IMPDH2,Interferon alpha,LGMN,LIG1, Mek,MHC Class I (complex),OPA1,OXCT1,PHB,PI3K (complex),Pro-inflammatory Cytokine,Raf,Rb, Rsk, Sos, STAT3, TARDBP,TMPO,Tnf receptor | 25 | 15 | Cellular Growth and Proliferation, Cell Death, Cell Cycle |
| 6 | ATP2A2,Calcineurin protein(s),Calpain,Camk, CAMK2G, CaMKII, CANX,DAD1 (includes EG:13135),DLG1,DOCK7, Fibrinogen, GNB2L1,Ikk (family),Integrin,Integrin alpha 2 beta 1, Integrin alpha 3 beta 1,Integrin alpha 4 beta 1,Integrin alpha 5 beta 1,Integrin alpha 5 beta 3,Integrinα, ITGA1, ITGA2, ITGA5,KCND3,Laminin,Lfa-1,MAP1A, MAP2K1/2, NFkB (complex), PICALM,Pmca,SSR1,STMN1,Talin,Vla-4 | 24 | 15 | Cell Morphology, Cell-To-Cell Signaling and Interaction, Tissue Development |
| 7 | 14-3-3,AMPK,Ap1,CD3,CROT,CS,DDX4,Focal adhesion kinase,hCG,HDLBP,Histone h3,IL1,Insulin,Jnk,Lh, LYPLA1, Mmp, MYLPF,P38 MAPK,p85 (pik3r), Pkc(s), PTGIS, Rac, Ras, Ras homolog, SEC23A, SEC31A,SPTAN1, SPTBN4, TCR, Try4, Try5,Trypsin,UQCRC2,Vegf | 22 | 14 | Gene Expression, Cell Death, Cell Morphology |
| 8 | ACSL4,ALDH4A1,Arf,AURKB,CSTF1,DCAF4,DDB1,DDB2,DFFB,DOCK7,EIF2AK2,EIF2B1,FGF1,G3BP1,GLG1 (includes EG:20340), Ifn gamma,LRRC17,mir-30,miR-30c/miR-30a/miR-30d (includes others),MURC, MYC, NCEH1, NPM1,PDX1 (includes EG:18609),PTGES3,SART1, SEC62 (includes EG:294912),SNRNP27, SNRNP200,TP53 (includes EG:22059),TRIP12,TXNL4A,USP7,USP39,UTP15 | 22 | 14 | DNA Replication, Recombination, and Repair, Cancer, Skeletal and Muscular Disorders |
| 9 | APP,CALCOCO1,CCND1,COL1A1,EIF2AK2,EPG5,ERG,ERMP1,EVI5,FOS,GATA2,GLG1 (includes EG:20340), GRB2, IL16, IL24,IL32,IL12 (complex),IL4 (includes EG:16189), INF2,KCNIP3,MEIS1,Naca,PDXDC1,PEX14 (includes EG: 40294), progesterone,PTPN2,Rps24,RPS6KA3, Runx1, SEC23B, SELE (includes EG:20339),SHKBP1,SRF,Tnf,Tnf receptor | 19 | 13 | Hematological System Development and Function, Hematopoiesis, Cellular Development |
| 10 | CTSA,DDB2,DDX3Y,Ddx3y (mouse),DRG1,ENTPD2, ETFB, GPC4, GPD2,HIF1A,HMGCL,HSPD1,IDI1,IFN alpha/beta, IFN TYPE 1, IFNA16,Ifna,IFNB1 (includes EG:15977),IFNG (includes EG:15978), IFNK,IL12 (family),IRG,KANK2,L-triiodothyronine,LCK,MAVS,NUB1,PRKRA,PTPN2,RAB12,RB1,RIPK1,RNASEL,SPG21,TIRAP | 18 | 13 | Cellular Development, Hematopoiesis, Cell-To-Cell Signaling and Interaction |
| 11 | 26s Proteasome,Alpha catenin, ANXA11,AP1G2, ASB2, ASB9, BAG3, CLDN3,CUL5,EIF2AK2, ERBB3,EXOC4, FSH, G-protein beta,HISTONE,Hsp70,IDH2,Ifn,IFNA16, Ifna, IFNK,Ikb,IkB-NfkB,Mapk,MAVS,NUB1,Pdgfr, Pka, PRKACA, PRKRA,Shc,STAT,TIRAP,Ubiquitin,VPS28 (includes EG:300052) | 10 | 11 | Cell Death, Inflammatory Response, Infectious Disease |
