# Supplementary material for: Quantitative Proteomic Analysis of BHK-21 Cells Infected with Foot-and-Mouth Disease Virus Serotype Asia 1
Source: PLoS One. 2015 Jul 10;10(7):e0132384. doi: 10.1371/journal.pone.0132384 (PMC4498813; doi:10.1371/journal.pone.0132384)
Supplement: S3 Table — (DOCX) [file pone.0132384.s008.docx]

**S3 Table**.**Details of 11 Networks.**

| **Molecules in Network 1** | **Protein names** |
| --- | --- |
| Akt | Proline-rich AKT1 substrate 1 |
| ATP5A1 | ATP synthase subunit alpha, |
| ATP5D | ATP synthase, H+ transporting, mitochondrial F1 complex, delta subunit, isoform CRA_c |
| ATPAF2 | ATP synthase mitochondrial F1 complex assembly factor 2 |
| CDC5L | Cell division cycle 5-like protein |
| CHTOP | Chromatin target of PRMT1 protein |
| DARS | Aspartate--tRNA ligase, cytoplasmic |
| EIF1 | Eukaryotic translation initiation factor 1 |
| Eif2 | Eukaryotic translation initiation factor 2 subunit 2 |
| EIF1B | Eukaryotic translation initiation factor 1b |
| EIF2AK2 | Interferon-induced, double-stranded RNA-activated protein kinase |
| EIF2S3 | Eukaryotic translation initiation factor 2 subunit 3 |
| EIF4A3 | Eukaryotic initiation factor 4A-III |
| GPI | Glucose-6-phosphate isomerase |
| MTORC1 | Proline-rich AKT1 substrate 1 |
| RBM7 | RNA-binding protein 7 |
| Ribosomal 40s subunit | 40S ribosomal protein S2 |
| Rnr | Nuclear receptor subfamily 4 group A member 2 |
| RPS3 | 40S ribosomal protein S3 |
| RPS6 | 40S ribosomal protein S6 |
| RPS12 | 40S ribosomal protein S12 |
| RPS24 | 40S ribosomal protein S24 |
| RPS26 | 40S ribosomal protein S6 |
| RPS3A | 40S ribosomal protein S3A |
| SARNP | SAP domain-containing ribonucleoprotein |
| Scd3 | Fatty acid desaturase |
| Scd4 | Scd4 protein |
| SCD | Scavenger receptor cysteine-rich type 1 protein M130 |
| Spectrin | Spectrin alpha chain, brain |
| SPTB | Spectrin beta chain, erythrocyte |
| SPTBN1 | Spectrin beta chain, brain 1 |
| SPTBN2 | Spectrin beta chain, brain 2 |
| SRRM2 | Serine/arginine repetitive matrix protein 2 |
| stearoyl-CoA 9-desaturase | Acyl-CoA desaturase 1 |
| UPF1 | Regulator of nonsense transcripts 1 |

| **Molecules in Network 2** | **Protein names** |
| --- | --- |
| Actin | Actin-like protein 7A,Actin-like protein 7B |
| ARPC2 | Actin-related protein 2/3 complex subunit 2 |
| ATPase | Transitional endoplasmic reticulum ATPase |
| BTF3 | Transcription factor BTF3 |
| Calmodulin | Abnormal spindle-like microcephaly-associated protein homolog |
| CD44 (includes EG:100330801) | CD44 antigen |
| Ck2 | Casein kinase II subunit beta (CK II beta) (Phosvitin) |
| EDF1 | Endothelial differentiation-related factor 1 |
| ERK | Mitogen-activated protein kinase 3 |
| Estrogen Receptor | Estrogen Receptor |
| F Actin | Actin-like protein |
| Holo RNA polymerase II | General transcription factor IIH subunit 2 |
| Hsp90 | Hsp90 co-chaperone Cdc37-like 1 |
| MYH1 | Myosin-1 |
| MYH2 | Myosin-2 |
| MYH3 | Myosin-3 |
| MYH4 | Myosin-4 |
| MYH6 | Myosin-6 |
| MYH7 | Myosin-7 |
| MYH8 | Myosin-8 |
| Myosin | Protein phosphatase 1 regulatory subunit 12A |
| MYRIP | Rab effector MyRIP |
| NEDD4 | E3 ubiquitin-protein ligase NEDD4 |
| PDCD6IP | Programmed cell death 6-interacting protein |
| PLS1 | Plastin-1 |
| POLR2C | DNA-directed RNA polymerase II subunit RPB3 |
| POLR2G | DNA-directed RNA polymerase II subunit RPB7 |
| RNA polymerase II | DNA-directed RNA polymerase II subunit RPB1 |
| SAFB2 | Scaffold attachment factor B2 |
| SAFB | Scaffold attachment factor B1 |
| SUGT1 | Suppressor of G2 allele of SKP1 homolog |
| TCEA1 | Transcription elongation factor A protein 1 |
| TIMM17B | Mitochondrial import inner membrane translocase subunit Tim17-B |
| TOMM70A | Mitochondrial import receptor subunit TOM70 |
| UBAP2L | Ubiquitin-associated protein 2-like |

| **Molecules in Network 3** | | **Protein names** | |
| --- | --- | --- | --- |
| C11orf58 | Chromosome 11 open reading frame 58 | |  |
| CAPRIN1 | Caprin1 protein | |  |
| CHCHD6 | Coiled-coil-helix-coiled-coil-helix domain-containing protein 6 | |  |
| CPSF7 | Cleavage and polyadenylation specificity factor subunit 7 | |  |
| CRIPT | Cysteine-rich PDZ-binding protein | |  |
| CUL5 | Cullin-5 | |  |
| DAD1 (includes EG:13135) | Dolichyl-diphosphooligosaccharide--protein glycosyltransferase subunit DAD1 | |  |
| DLG4 | Disks large homolog 4 | |  |
| DLST | Dihydrolipoyllysine-residue succinyltransferase component of 2-oxoglutarate dehydrogenase complex, mitochondrial | |  |
| DNAJC11 | DnaJ homolog subfamily C member 11 | |  |
| FXYD7 | FXYD domain-containing ion transport regulator 7 | |  |
| G3BP1 | RasGTPase-activating protein-binding protein 1 | |  |
| GPAA1 | Glycosylphosphatidylinositol anchor attachment 1 protein | |  |
| HNF4A | Hepatocyte nuclear factor 4-alpha | |  |
| IFI30 | Gamma-interferon-inducible lysosomalthiolreductase | |  |
| KRI1 | Protein KRI1 homolog | |  |
| MTMR2 | Myotubularin-related protein 2 | |  |
| MTX1 | Metaxin-1 | |  |
| MTX2 | Metaxin-2 | |  |
| NDFIP1 | NEDD4 family-interacting protein 1 | |  |
| NEDD4L | E3 ubiquitin-protein ligase NEDD4-like | |  |
| NOC3L | Nucleolar complex protein 3 homolog | |  |
| OGFOD1 | 2-oxoglutarate and iron-dependent oxygenase domain-containing protein 1 | |  |
| PGM3 | Phosphoacetylglucosaminemutase | |  |
| PGRMC2 | Membrane-associated progesterone receptor component 2 | |  |
| PIGS | GPI transamidase component PIG-S | |  |
| SAMM50 | Sorting and assembly machinery component 50 homolog | |  |
| SLIRP | SRA stem-loop-interacting RNA-binding protein, mitochondrial | |  |
| SSSCA1 | Sjoegren syndrome/scleroderma autoantigen 1 homolog | |  |
| TGFB1 (includes EG:21803) | Transforming growth factor beta-1 | |  |
| TOE1 | Target of EGR1 protein 1 | |  |
| UMPS | Uridine 5'-monophosphate synthase | |  |
| WDR36 | Wdr36 protein | |  |
| YKT6 | Synaptobrevin homolog YKT6 | |  |
| ZNF207 | Zinc finger protein 207, isoform CRA_c | |  |

| **Molecules in Network 4** | **Protein names** |
| --- | --- |
| Alp | Antileukoproteinase |
| ANXA5 | Annexin A5 |
| C1QBP | Complement component 1 Q subcomponent-binding protein, mitochondrial |
| CCDC6 | Coiled-coil domain-containing protein 6 |
| collagen | Collagen alpha-1(VII) chain |
| Collagen Alpha1 | Prolyl 4-hydroxylase subunit alpha-1,Collagen alpha-1(VII) chain |
| Collagen type I | Collagen alpha-1(VII) chain |
| Collagen type III | Collagen alpha-1(VII) chain |
| Collagen type IV | Collagen alpha-1(VII) chain |
| Collagen(s) | Collagen alpha-1(VII) chain |
| COPA | Copa protein |
| CTSL2 | CTD small phosphatase-like protein 2 |
| DNAJA3 | DnaJ homolog subfamily A member 3, mitochondrial |
| EIF4G2 | Eukaryotic translation initiation factor 4 gamma 2 |
| Eotaxin | Eotaxin |
| ERK1/2 | Chemokine-like receptor 1 |
| Fcer1 | Linker for activation of T-cells family member 2 |
| GBAS | Protein NipSnap homolog 2 |
| ITGB3 | Integrin beta-3 |
| Laminin1 | Laminin subunit beta-1 |
| LGALS3 | Galectin-3 |
| Mlc | Myosin light chain 4 |
| MRC2 | C-type mannose receptor 2 |
| MYL4 | Myosin light chain 4 |
| p70 S6k | Ribosomal protein S6 kinase beta-1 |
| Pdgf (complex) | Platelet-derived growth factor receptor beta |
| PDGF BB | Platelet-derived growth factor receptor beta |
| PLC gamma | 1-phosphatidylinositol-4,5-bisphosphate phosphodiesterase gamma-1 |
| PP2A | Serine/threonine-protein phosphatase 2A catalytic subunit alpha isoform |
| PRKAA1 | 5'-AMP-activated protein kinase catalytic subunit alpha-1 |
| RPL23A | 60S ribosomal protein L23a |
| RPTOR | Regulatory-associated protein of mTOR |
| SLC25A11 | Mitochondrial 2-oxoglutarate/malate carrier protein |
| Tgf beta | TGF-beta receptor type-2 |
| TIMP1 | Metalloproteinase inhibitor 1 |

| **Molecules in Network 5** | **Protein names** |
| --- | --- |
| BCR | Bcr protein |
| BSG (includes EG:12215) | Basigin |
| Caspase | DNA fragmentation factor subunit beta |
| CUL3 (includes EG:26554) | Cullin-3 |
| Cyclin A | Cyclin-A |
| Cyclin E | Cyclin-E |
| Cytochrome c | Cytochrome c1, heme protein, mitochondrial |
| DDX5 | Probable ATP-dependent RNA helicase DDX5 |
| DDX17 | Probable ATP-dependent RNA helicase DDX17 |
| DDX3X | ATP-dependent RNA helicase DDX3X |
| E2f | Transcription factor E2F |
| HRAS | GTPaseHRas |
| IFN Beta | Interferon beta |
| Ifn gamma | Interferon gamma receptor 1 |
| Igm | B-cell antigen receptor complex-associated protein alpha chain |
| Immunoglobulin | Leukocyte immunoglobulin-like receptor subfamily B member 3 |
| IMPDH2 | Inosine-5'-monophosphate dehydrogenase 2 |
| Interferon alpha | Interferon alpha-1 |
| LGMN | Legumain |
| LIG1 | Leucine-rich repeats and immunoglobulin-like domains protein 1 |
| Mek | Dual specificity mitogen-activated protein kinase kinase 1 |
| MHC Class I (complex) | Major histocompatibility complex class I-related gene protein |
| OPA1 | Dynamin-like 120 kDa protein, mitochondrial |
| OXCT1 | Succinyl-CoA:3-ketoacid-coenzyme A transferase 1, mitochondrial |
| PHB | Para-hydroxybenzoate--polyprenyltransferase |
| PI3K (complex) | Phosphatidylinositol-4,5-bisphosphate 3-kinase catalytic subunit gamma isoform |
| Pro-inflammatory Cytokine | Tax1-binding protein 1 homolog |
| Raf | RAF proto-oncogene serine/threonine-protein kinase |
| Rb | Retinoblastoma-associated protein |
| Rsk | Ribosomal protein S6 kinase alpha-3 |
| Sos | Son of sevenless homolog 1 |
| STAT3 | Stat3 protein |
| TARDBP | TAR DNA-binding protein 43 |
| TMPO | Lamina-associated polypeptide 2, isoforms beta/delta/epsilon/gamma |
| Tnf receptor | Tumor necrosis factor |

| **Molecules in Network 6** | **Protein names** |
| --- | --- |
| ATP2A2 | Sarcoplasmic/endoplasmic reticulum calcium ATPase 2 |
| Calcineurin protein(s) | Calcipressin-1 |
| Calpain | Calpain-2 catalytic subunit |
| Camk | Calcium/calmodulin-dependent protein kinase type IV |
| CAMK2G | Calcium/calmodulin-dependent protein kinase type II subunit gamma |
| CaMKII | Calcium/calmodulin-dependent protein kinase type II subunit delta |
| CANX | Calnexin |
| DAD1 (includes EG:13135) | Dolichyl-diphosphooligosaccharide--protein glycosyltransferase subunit DAD1 |
| DLG1 | Disks large homolog 1 |
| DOCK7 | Dedicator of cytokinesis protein 7 |
| Fibrinogen | Fibrinogen gamma chain |
| GNB2L1 | Guanine nucleotide-binding protein subunit beta-2-like 1 |
| Ikk (family) | Inhibitor of nuclear factor kappa-B kinase subunit beta |
| Integrin | Integrin alpha-7 |
| Integrin alpha 2 beta 1 | Integrin alpha-7 |
| Integrin alpha 3 beta 1 | Integrin alpha-3 |
| Integrin alpha 4 beta 1 | Integrin alpha-4 |
| Integrin alpha 5 beta 1 | Integrin beta-5 |
| Integrin alpha 5 beta 3 | Integrin alpha-3 |
| Integrinα | Integrin-alpha FG-GAP repeat-containing protein 2 |
| ITGA1 | Integrin alpha-1 |
| ITGA2 | Integrin alpha-2 |
| ITGA5 | Integrin alpha-5 |
| KCND3 | Potassium voltage-gated channel subfamily D member 3 |
| Laminin | Laminin subunit gamma-1 |
| Lfa-1 | Integrin alpha-L |
| MAP1A | Microtubule-associated protein 1A |
| MAP2K1/2 | Interleukin-17 receptor D |
| NFkB (complex) | Tumor necrosis factor receptor superfamily member 11A |
| PICALM | Phosphatidylinositol-binding clathrin assembly protein |
| Pmca | Plasma membrane calcium-transporting ATPase 2 |
| SSR1 | Translocon-associated protein subunit alpha |
| STMN1 | Stathmin |
| Talin | Talin-1 |
| Vla-4 | Integrin beta-1 |

| **Molecules in Network 7** | **Protein names** |
| --- | --- |
| 14-3-3 | 14-3-3 protein eta |
| AMPK | 5'-AMP-activated protein kinase subunit gamma-2 |
| Ap1 | Transcription factor AP-1 |
| CD3 | T-cell surface glycoprotein CD3 zeta chain |
| CROT | Peroxisomalcarnitine O-octanoyltransferase |
| CS | Citrate synthase, mitochondrial |
| DDX4 | Probable ATP-dependent RNA helicase DDX4 |
| Focal adhesion kinase | Focal adhesion kinase 1 |
| hCG | Ubiquitin-like protein 3 |
| HDLBP | Vigilin |
| Histone h3 | Histone H3.2 |
| IL1 | Interleukin-1 beta |
| Insulin | Insulin-like peptide INSL5 |
| Jnk | C-Jun-amino-terminal kinase-interacting protein 3 |
| Lh | Procollagen-lysine,2-oxoglutarate 5-dioxygenase 2 |
| LYPLA1 | Acyl-protein thioesterase 1 |
| Mmp | Matrix metalloproteinase-14 |
| MYLPF | Myosin regulatory light chain 2, skeletal muscle isoform |
| P38 MAPK | Mitogen-activated protein kinase 14 |
| p85 (pik3r) | Phosphatidylinositol 3-kinase regulatory subunit alpha |
| Pkc(s) | Protein kinase C alpha type |
| PTGIS | Prostacyclin synthase |
| Rac | FYVE, RhoGEF and PH domain-containing protein 1 |
| Ras | Ras-specific guanine nucleotide-releasing factor 2 |
| Ras homolog | Ras-specific guanine nucleotide-releasing factor 1 |
| SEC23A | Protein transport protein Sec23A |
| SEC31A | Protein transport protein Sec31A |
| SPTAN1 | Spectrin alpha chain, brain |
| SPTBN4 | Disrupted in schizophrenia 1 homolog |
| TCR | Sperm motility kinase Tcr mutant form |
| Try4 | Trypsin-4 |
| Try5 | Trypsin 5 |
| Trypsin | Kunitz-type protease inhibitor 4 |
| UQCRC2 | Cytochrome b-c1 complex subunit 2, mitochondrial |
| Vegf | Vascular endothelial growth factor A |

| **Molecules in Network 8** | **Protein names** |
| --- | --- |
| ACSL4 | Long-chain-fatty-acid--CoA ligase 4 |
| ALDH4A1 | Delta-1-pyrroline-5-carboxylate dehydrogenase, mitochondrial |
| Arf | ADP-ribosylation factor GTPase-activating protein 1 |
| AURKB | Aurora kinase B |
| CSTF1 | Cleavage stimulation factor subunit 1 |
| DCAF4 | Cullin-4A |
| DDB1 | Protein VPRBP |
| DDB2 | DNA damage-binding protein 2 |
| DFFB | DNA fragmentation factor subunit beta |
| DOCK7 | Dedicator of cytokinesis protein 7 |
| EIF2AK2 | Interferon-induced, double-stranded RNA-activated protein kinase |
| EIF2B1 | Translation initiation factor eIF-2B subunit alpha |
| FGF1 | Fibroblast growth factor 1 |
| G3BP1 | RasGTPase-activating protein-binding protein 1 |
| GLG1 (includes EG:20340) | Golgi apparatus protein 1 |
| Ifn gamma | Interferon gamma receptor 1 |
| LRRC17 | Leucine-rich repeat-containing protein 17 |
| mir-30 | E3 ubiquitin-protein ligase MARCH8 |
| miR-30c/miR-30a/miR-30d (includes others) | E3 ubiquitin-protein ligase MARCH8 |
| MURC | Muscle-related coiled-coil protein |
| MYC | Myc proto-oncogene protein |
| NCEH1 | Neutral cholesterol ester hydrolase 1 |
| NPM1 | Nucleophosmin |
| PDX1 (includes EG:18609) | Speckle-type POZ protein |
| PTGES3 | Prostaglandin E synthase 3 |
| SART1 | U4/U6.U5 tri-snRNP-associated protein 1 |
| SEC62 (includes EG:294912) | Translocation protein SEC62 |
| SNRNP27 | U4/U6.U5 small nuclear ribonucleoprotein 27 kDa protein |
| SNRNP200 | Pre-mRNA-processing-splicing factor 8 |
| TP53 (includes EG:22059) | TP53-regulating kinase |
| TRIP12 | Trip12 protein |
| TXNL4A | Thioredoxin-like protein 4A |
| USP7 | Ubiquitin carboxyl-terminal hydrolase 7 |
| USP39 | U4/U6.U5 tri-snRNP-associated protein 2 |
| UTP15 | U3 small nucleolar RNA-associated protein 15 homolog |

| **Molecules in Network 9** | **Protein names** |
| --- | --- |
| APP | Amyloid beta A4 protein |
| CALCOCO1 | Calcium-binding and coiled-coil domain-containing protein 1 |
| CCND1 | G1/S-specific cyclin-D1 |
| COL1A1 | Collagen alpha-1(I) chain (Alpha-1 type I collagen) |
| EIF2AK2 | Interferon-induced, double-stranded RNA-activated protein kinase |
| EPG5 | Ectopic P granules protein 5 homolog |
| ERG | Transcriptional regulator ERG |
| ERMP1 | Endoplasmic reticulum metallopeptidase 1 |
| EVI5 | Ecotropic viral integration site 5 protein |
| FOS | Proto-oncogene c-Fos |
| GATA2 | Endothelial transcription factor GATA-2 |
| GLG1 (includes EG:20340) | Golgi apparatus protein 1 |
| GRB2 | Growth factor receptor-bound protein 2 |
| IL16 | Pro-interleukin-16 |
| IL24 | Interleukin-24 |
| IL32 | Interleukin-32 |
| IL12 (complex) | Interleukin-12 receptor subunit beta-1 |
| IL4 (includes EG:16189) | Interleukin-4 |
| INF2 | Inverted formin-2 |
| KCNIP3 | Calsenilin |
| MEIS1 | Homeobox protein Meis1 |
| Naca | Nascent polypeptide-associated complex subunit alpha |
| PDXDC1 | Pyridoxal-dependent decarboxylase domain-containing protein 1 |
| PEX14 (includes EG:40294) | Peroxisomal membrane protein PEX14 |
| progesterone | Membrane-associated progesterone receptor component 1 |
| PTPN2 | Tyrosine-protein phosphatase non-receptor type 2 |
| Rps24 | 40S ribosomal protein S24 |
| RPS6KA3 | Ribosomal protein S6 kinase alpha-3 |
| Runx1 | Runt-related transcription factor 1 |
| SEC23B | Protein transport protein Sec23B |
| SELE (includes EG:20339) | E-selectin |
| SHKBP1 | SH3KBP1-binding protein 1 |
| SRF | Serum response factor |
| Tnf | Tumor necrosis factor |
| Tnf receptor | Tumor necrosis factor |

| **Molecules in Network 10** | **Protein names** |
| --- | --- |
| CTSA | Lysosomal protective protein |
| DDB2 | Ddb2 protein |
| DDX3Y | ATP-dependent RNA helicase DDX3Y |
| Ddx3y (mouse) | ATP-dependent RNA helicase DDX3Y |
| DRG1 | Developmentally-regulated GTP-binding protein 1 |
| ENTPD2 | Ectonucleoside triphosphate diphosphohydrolase 2 |
| ETFB | Electron transfer flavoprotein subunit beta |
| GPC4 | Glypican-4 |
| GPD2 | Glycerol-3-phosphate dehydrogenase, mitochondrial |
| HIF1A | Hypoxia-inducible factor 1-alpha |
| HMGCL | Hydroxymethylglutaryl-CoA lyase, mitochondrial |
| HSPD1 | 60 kDa heat shock protein, mitochondrial |
| IDI1 | Isopentenyl-diphosphate Delta-isomerase 1 |
| IFN alpha/beta | Interferon alpha/beta receptor 2 |
| IFN TYPE 1 | Interferon gamma receptor 1 |
| IFNA16 | Interferon alpha-16 |
| Ifna | Interferon alpha-1 |
| IFNB1 (includes EG:15977) | Interferon beta |
| IFNG (includes EG:15978) | Interferon gamma |
| IFNK | Interferon kappa |
| IL12 (family) | Interleukin-12 receptor subunit beta-1 |
| IRG | Interferon-induced protein with tetratricopeptide repeats 3 |
| KANK2 | KN motif and ankyrin repeat domain-containing protein 2 |
| L-triiodothyronine | Sulfotransferase 4A1 |
| LCK | Proto-oncogene tyrosine-protein kinase LCK |
| MAVS | Mitochondrial antiviral-signaling protein |
| NUB1 | NEDD8 ultimate buster 1 |
| PRKRA | Interferon-inducible double stranded RNA-dependent protein kinase activator A |
| PTPN2 | Tyrosine-protein phosphatase non-receptor type 2 |
| RAB12 | Ras-related protein Rab-12 |
| RB1 | RNA-binding protein 45 |
| RIPK1 | Receptor-interacting serine/threonine-protein kinase 1 |
| RNASEL | 2-5A-dependent ribonuclease |
| SPG21 | Maspardin |
| TIRAP | Toll/interleukin-1 receptor domain-containing adapter protein |

| **Molecules in Network 11** | **Protein names** |
| --- | --- |
| 26s Proteasome | Probable 26S proteasome non-ATPase regulatory subunit 3a |
| Alpha catenin | Catenin alpha-2 |
| ANXA11 | annexin A11 |
| AP1G2 | AP-1 complex subunit gamma-like 2 |
| ASB2 | Ankyrin repeat and SOCS box protein 2 |
| ASB9 | Ankyrin repeat and SOCS box protein 9 |
| BAG3 | BAG family molecular chaperone regulator 3 |
| CLDN3 | Claudin-3 |
| CUL5 | Cullin-5 |
| EIF2AK2 | Eukaryotic translation initiation factor 2-alpha kinase 2 |
| ERBB3 | Receptor tyrosine-protein kinase erbB-3 |
| EXOC4 | Exocyst complex component 4 |
| FSH | Follitropin subunit beta |
| G-protein beta | C-C chemokine receptor type 7 |
| HISTONE | Histone acetyltransferase KAT2B |
| Hsp70 | Heat shock 70 kDa protein 1A |
| IDH2 | Isocitrate dehydrogenase [NADP], mitochondrial |
| Ifn | Interferon beta |
| IFNA16 | Interferon alpha-16 |
| Ifna | Ifna6 protein |
| IFNK | Interferon kappa |
| Ikb | NF-kappa-B essential modulator |
| IkB-NfkB | NF-kappa-B-repressing factor |
| Mapk | Mitogen-activated protein kinase 1 |
| MAVS | Mitochondrial antiviral-signaling protein |
| NUB1 | Nub1 protein |
| Pdgfr | Platelet-derived growth factor receptor beta |
| Pka | cAMP-dependent protein kinase catalytic subunit alpha |
| PRKACA | cAMP-dependent protein kinase catalytic subunit alpha |
| PRKRA | Interferon-inducible double stranded RNA-dependent protein kinase activator A |
| Shc | SHC-transforming protein 3 |
| STAT | Suppressor of cytokine signaling 1 |
| TIRAP | Toll/interleukin-1 receptor domain-containing adapter protein |
| Ubiquitin | Ubiquitin-like modifier-activating enzyme 5 |
| VPS28(includes EG:300052) | Vacuolar protein sorting-associated protein 28 homolog |
